# Nanocarrier strategies to overcome P-glycoprotein-mediated drug resistance in cancer therapy

**DOI:** 10.3762/bjnano.17.64

**Published:** 2026-07-13

**Authors:** Andreina Quevedo-Enríquez, Katty Yi Zhang, Denisse Yajaira Enriquez, Byron Raul Inapanta, Roxana Noemí Peroni, Christian Rafael Quijia

**Affiliations:** 1 Institute of Pharmacological Research (ININFA UBA-CONICET), Faculty of Pharmacy and Biochemistry, University of Buenos Aires, Junín 956 5° 1113, Autonomous City of Buenos Aires, Argentina; 2 School of Biological Sciences and Engineering, Yachay Tech University, Hda. San José s/n y Proyecto Yachay, Urcuquí 100119, Ecuador; 3 Chair of Pharmacology, Department of Pharmacology, Faculty of Pharmacy and Biochemistry, University of Buenos Aires, Junín 956 5°, 1113, Autonomous City of Buenos Aires, Argentina

**Keywords:** cancer chemotherapy, efflux transporters, multidrug resistance (MDR), nanoparticles, siRNA nanocarriers, tumor-targeted therapy

## Abstract

Multidrug resistance (MDR) remains a major barrier to successful cancer chemotherapy, frequently resulting in therapeutic failure, tumor relapses, and poor clinical outcomes. Among the diverse mechanisms underlying MDR, the overexpression of ATP-binding cassette (ABC) transporters, particularly P-glycoprotein (P-gp, encoded by ABCB1) is one of the most extensively studied as it actively effluxes structurally diverse chemotherapeutic agents and reduces intracellular drug exposure below cytotoxic thresholds. In this review, we critically examine recent nanocarrier-based strategies developed to overcome P-gp-mediated resistance across major malignancies, including breast, lung, colorectal, gastric, and prostate cancers. These approaches are categorized according to their principal mechanisms of action: (i) direct functional inhibition of P-gp ATPase activity using small-molecule modulators such as quercetin, ᴅ-α-tocopheryl polyethylene glycol succinate, and tariquidar, (ii) circumvention of membrane efflux through receptor-mediated endocytosis, intracellular trafficking control, or tumor-responsive drug release, and (iii) suppression of transporter expression via co-delivery of siRNA, shRNA, or anti-miRNA payloads targeting ABCB1 regulatory pathways. We further discuss advances in nanoplatform engineering, including lipid-based nanoparticles, polymeric micelles, lipid–polymer hybrid systems, and biomimetic carriers designed to enhance tumor selectivity and intracellular retention. Preclinical evidence consistently demonstrates improved drug accumulation, restored chemosensitivity, and reduced systemic toxicity. Nevertheless, clinical translation remains constrained by tumor heterogeneity, variable biological barriers, large-scale manufacturing requirements, and regulatory complexity. Overall, nanoparticle-mediated modulation of P-gp represents a promising strategy toward precision oncology, although future success will depend on scalable design, mechanistic standardization, and biomarker-guided clinical implementation.

## Review

### 1 Introduction

#### 1.1 Cancer and current challenges in chemotherapy

Cancer remains one of the leading causes of mortality worldwide, with millions of new cases diagnosed annually. Among the most prevalent and clinically challenging malignancies are breast, colorectal, gastric, prostate, and lung cancers, particularly non-small cell lung cancer (NSCLC). Despite advances in early detection and treatment, the long-term effectiveness of chemotherapy is frequently compromised by multidrug resistance (MDR), a phenomenon in which cancer cells evade the cytotoxic effects of drugs through various mechanisms. Overexpression of efflux transporters, particularly P-glycoprotein (P-gp), is a well-documented contributor to this phenotype, although it is not the sole mechanism involved. This review focuses specifically on P-gp-mediated resistance and nanocarrier-based strategies developed to circumvent it.

**1.1.1 Breast cancer.** Breast cancer is the most often diagnosed cancer in women and the leading cause of cancer-related death in the female population [1]. It is characterized by uncontrolled proliferation of breast tissue cells and marked clinical heterogeneity. Among the resistance mechanisms documented in breast cancer, overexpression of P-gp has been studied extensively as a factor that actively exports chemotherapeutic agents from cancer cells, reducing intracellular concentration and therapeutic effectiveness [2]. Surmounting P-gp-mediated resistance has therefore emerged as a goal in experimental breast cancer research.

**1.1.2 Colorectal cancer.** Colorectal cancer, affecting the colon and rectum, ranks among the most prevalent cancers worldwide and significantly contributes to cancer-related mortality. The World Health Organization reported about 1.9 million new cases and more than 930,000 deaths in 2020, with marked geographical variation in incidence and mortality [3]. A major obstacle in its therapy is MDR, often linked to ABC transporters including P-glycoprotein (P-gp, ABCB1), multidrug resistance-associated protein 1 (MRP1, ABCC1), and breast cancer resistance protein (BCRP, ABCG2) [4]. Because this review focuses on P-gp-mediated MDR, ABCB1 overexpression and its downstream consequences for intracellular drug concentration are the primary focus here; MRP1 and BCRP are noted as parallel mechanisms that fall outside the scope.

**1.1.3 Gastric cancer.** Gastric cancer remains one of the most lethal malignancies, particularly prevalent in East Asia and Latin America. Although early-stage detection can lead to curative outcomes, advanced gastric cancer is frequently refractory to conventional chemotherapy regimens [5]. P-gp-mediated efflux constitutes a significant therapeutic barrier in this context, and the complex tumor microenvironment (TME), partly characterized by acidic gastric conditions, further complicates drug delivery and intracellular retention [6].

**1.1.4 Lung cancer and non-small cell lung cancer.** Lung cancer is the leading cause of cancer deaths globally, with NSCLC accounting for approximately 85% of cases. Despite the availability of targeted therapies and immunotherapies, chemotherapy remains an essential component of many treatment regimens [7–8]. P-gp overexpression, among other mechanisms, contributes to reduced long-term drug efficacy in NSCLC, and the high mutation rate and intratumoral heterogeneity characteristic of this disease create additional resistance barriers beyond efflux-mediated pathways [8].

**1.1.5 Prostate cancer.** Prostate cancer is the second most frequently diagnosed cancer in men worldwide. While early-stage prostate cancer can often be managed with hormone therapy or surgery, advanced disease often requires chemotherapy, which frequently becomes ineffective because of acquired MDR mechanisms [9]. In castration-resistant prostate cancer (CRPC), P-gp upregulation, operating alongside androgen receptor-mediated survival signaling, which can indirectly modulate ABCB1 expression via transcriptional co-regulatory pathways, contributes to chemoresistance, thereby motivating the development of strategies aimed at restoring drug sensitivity [10].

#### 1.2 Mechanisms of multidrug resistance in cancer

Multidrug resistance and metastasis are the main causes of cancer treatment failure. MDR tends to occur in cancer patients and is the ability of tumor cells to develop a specific mechanism to resist and overcome the cytotoxic or inhibitory effect of drug treatment, leading to a reduction in the efficacy of chemotherapy and to cancer progression [11]. Critically, resistance in solid tumors is typically multifactorial. Target alteration, pathway reconfiguration, defects in apoptotic signaling, enhanced DNA repair, epithelial-to-mesenchymal transition (EMT), and microenvironmental barriers all contribute alongside efflux-mediated mechanisms, depending on tumor type and treatment history. This review does not aim to encompass all these processes; rather, it focuses on P-gp-mediated efflux as one important and clinically significant mechanism, with nanocarrier-based interventions directed specifically at overcoming it.

MDR is classified as either intrinsic (primary) or acquired, according to the temporal relationship between resistance and drug exposure. Intrinsic resistance is governed by endogenous factors, including genetic and epigenetic alterations within tumor cells, as well as structural features of the TME present prior to any therapeutic challenge. Acquired resistance, by contrast, emerges following exposure to chemotherapeutic agents and reflects selective pressure exerted by treatment itself [12–13]. In both forms, tumors become refractory to structurally and functionally diverse drugs, which defines the clinical challenge of MDR.

Several molecular mechanisms associated with MDR in experimental models, including mutations that generate alternative pathways to preserve the cell cycle, inhibition of apoptosis, drug detoxification, drug sequestration by lysosomes, overexpression of efflux transporters, and enhanced DNA repair, have become obstacles to cancer therapy [14–16]. Among these, elevated drug efflux via cell membrane transporters, particularly P-glycoprotein, has been extensively documented as an important and common mechanism of chemoresistance across multiple tumors types [17].

**Figure 1:**
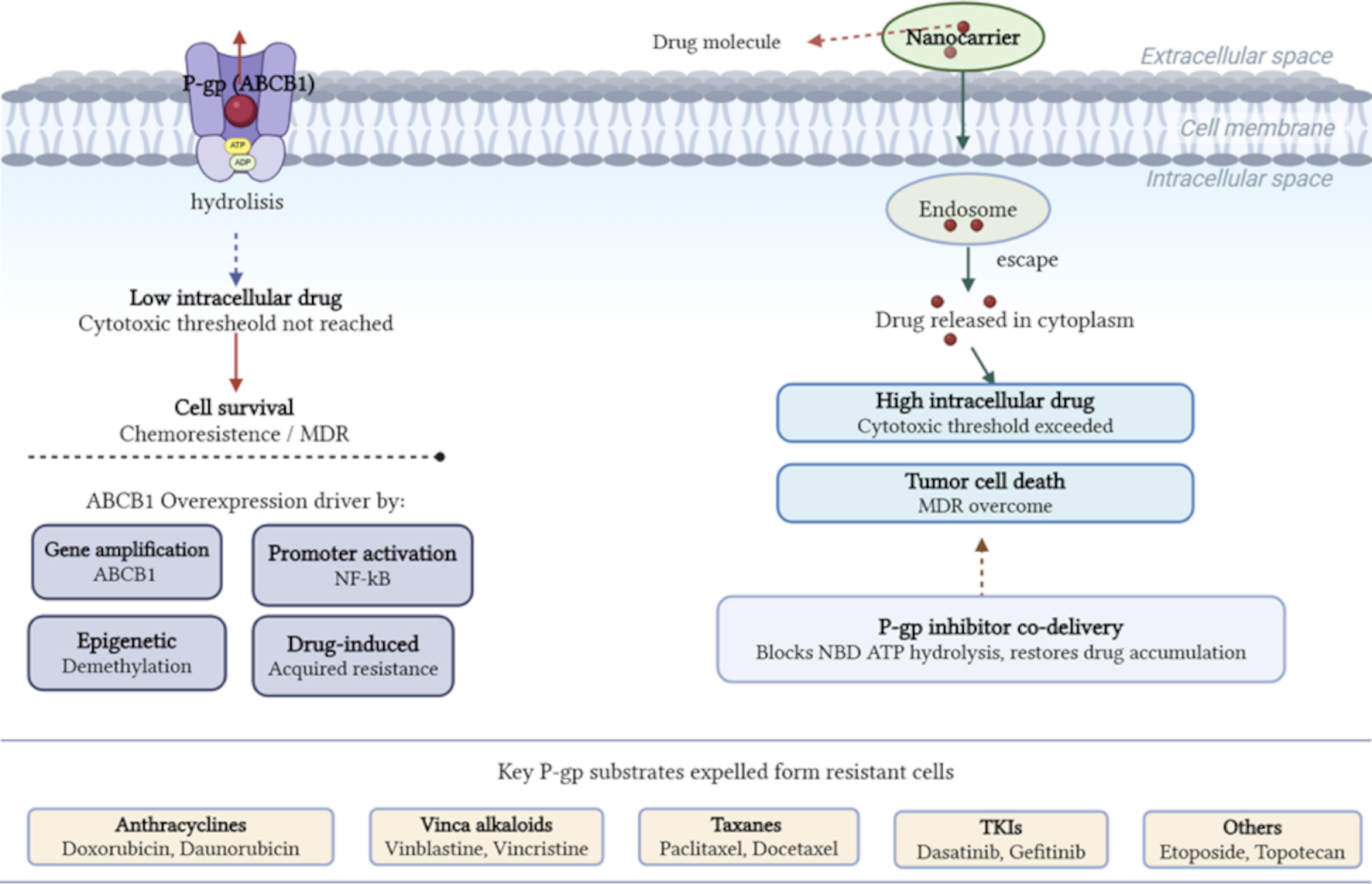
P-gp (ABCB1) expels hydrophobic chemotherapeutics via ATP hydrolysis (left), limiting intracellular drug accumulation and driving MDR. Nanocarriers bypass P-gp through endocytic uptake and may co-deliver inhibitors targeting the nucleotide-binding domains (NBD) (right), restoring cytotoxic efficacy.

**1.2.1 Non-P-gp MDR mechanisms that intersect with nanocarrier strategies.** Several resistance mechanisms not directly mediated by ABCB1 are nonetheless relevant to nanocarrier design. Lysosomal drug sequestration reduces the cytosolic availability of chemotherapeutics independently of P-gp, but pH-responsive nanocarriers engineered for endosomal escape indirectly address this barrier. EMT-associated phenotypic shifts alter membrane composition and uptake pathways in ways that affect nanoparticle internalization efficiency. Defects in apoptotic signaling, while downstream of drug–target interaction, can be partially circumvented by nanocarriers co-delivering pro-apoptotic agents alongside cytotoxic agents. These intersections are noted where relevant throughout the review, but mechanistic detail on non-ABCB1 pathways falls outside its primary scope. A schematic overview of P-gp-mediated resistance and nanocarrier-based strategies to overcome it is provided in Figure 1.

#### 1.3 P-glycoprotein as a therapeutic target

The ATP-binding cassette (ABC) superfamily comprises proteins that couple ATP binding and hydrolysis to the transmembrane movement of a broad range of substrates, including lipids, amino acids, ions, toxins, and pharmacological agents [18–19]. Within this family, three members have been characterized specifically in the context of chemoresistance, namely, ABCB1 (P-glycoprotein/MDR1), ABCG2 (breast cancer resistance protein, BCRP), and ABCC1 (multidrug resistance-associated protein 1, MRP1) [16,20].

P-glycoprotein, the first ABC transporter identified in relation to drug resistance, is the central subject of this review. Encoded by the ABCB1 gene and overexpressed across multiple cancer types, P-gp functions as an ATP-dependent efflux pump with broad substrate promiscuity for hydrophobic and amphipathic compounds. Clinically relevant chemotherapeutic agents expelled by P-gp include anthracyclines (doxorubicin, daunorubicin), vinca alkaloids (vinblastine, vincristine), taxanes (paclitaxel, docetaxel), and several tyrosine kinase inhibitors (dasatinib, gefitinib), among others [18,21]. The consequence in each case is a reduction in intracellular drug concentration sufficient to prevent the cytotoxic threshold from being reached. Understanding P-gp’s functional regulation and structural dynamics, from its nucleotide-binding domains to the conformational changes that drive substrate translocation, is therefore foundational to designing strategies that durably overcome MDR [14].

Escalating chemotherapeutic doses or increasing administration frequency are not viable solutions to P-gp-mediated resistance: Both approaches elevate systemic toxicity without reliably restoring drug sensitivity and are associated with decreased survival in patients with breast, colorectal, lung, and other cancers [18]. Co-administration of two or more synergistic drugs has offered partial improvements in chemotherapeutic efficacy, but the risk of off-target effects in healthy tissues remains a meaningful limitation [22–23]. The tissue distribution of P-gp further complicates systemic pharmacological inhibition. ABCB1 is expressed at high levels in tissues with excretory or barrier functions, including the liver, kidney, the apical surface of ileal and colonic epithelial cells, and the blood–brain barrier, while its expression is comparatively lower in the jejunum, duodenum, and stomach [22,24]. Systemic P-gp inhibitors, therefore, risk disrupting normal physiological efflux at these sites, a liability that has contributed to the clinical failure of several generations of pharmacological P-gp inhibitors.

Against this backdrop, nanoparticle-based drug delivery has emerged as a mechanistically distinct strategy. By encapsulating chemotherapeutic agents within nanocarriers that exploit endocytic uptake pathways, bypassing P-gp at the plasma membrane, and by enabling co-delivery of P-gp inhibitors or siRNA at therapeutically relevant intracellular concentrations, nanocarrier systems offer a route to restoring drug sensitivity without the systemic toxicity liabilities associated with conventional inhibition strategies [25].

### 2 Review methodology

This study is structured as a narrative review focusing on recent nanotechnological strategies developed to overcome MDR in cancer therapy. Particular emphasis was placed on systems targeting the P-glycoprotein efflux transporter. The objective was to compile and critically assess studies on inhibiting, bypassing, or modulating P-glycoprotein-mediated drug resistance.

#### 2.1 Search methodology

A non-systematic literature search was conducted using PubMed, Scopus, Web of Science, and Google Scholar databases. The search strategy was based on combinations of relevant keywords, including P-glycoprotein, multidrug resistance (MDR), nanoparticles, drug delivery, cancer, siRNA, efflux transporters, tumor microenvironment, stimuli-responsive systems, liposomes, polymeric micelles, hybrid nanocarriers, P-gp inhibitors, and cell membrane-coated nanoparticles. The search prioritized articles published between 2018 and 2025, further prioritizing original research articles, preclinical in vitro and in vivo studies, and relevant review papers.

#### 2.2 Search strategy and reproducibility

To improve transparency and methodological clarity, representative Boolean search strings were constructed and applied across the selected databases. The following combinations were used: (“P-glycoprotein” OR “ABCB1” OR “P-gp”) AND (“multidrug resistance” OR “MDR”) AND (“nanoparticles” OR “nanocarriers” OR “drug delivery systems”) AND (“cancer” OR “tumor”) and (“P-gp inhibitors” OR “efflux transporters”) AND (“liposomes” OR “polymeric micelles” OR “hybrid nanoparticles” OR “stimuli-responsive systems”) AND (“drug resistance”). These search strings were adapted to the syntax requirements of each database to optimize retrieval while maintaining specificity. The final literature search was conducted in March 2025.

#### 2.3 Criteria for inclusion and exclusion

Inclusion criteria were (i) studies focused on nanoparticle-based strategies targeting P-glycoprotein-mediated multidrug resistance in cancer, (ii) experimental evidence demonstrating MDR reversal (e.g., increased drug accumulation, reduced IC_50_ values, enhanced apoptosis), and (iii) peer-reviewed articles published in English.

Exclusion criteria were (i) studies not related to cancer therapy, (ii) articles lacking mechanistic insight or relevant experimental evidence to P-glycoprotein/MDR, and (iii) studies focused exclusively on conventional chemotherapy without nanotechnology.

#### 2.4 Screening and data selection process

All retrieved records from the selected databases were combined, and duplicate entries were removed manually and using reference management tools. A two-stage screening process was applied including (i) title and abstract screening to exclude studies unrelated to cancer, nanotechnology, or multidrug resistance and (ii) full-text evaluation to confirm relevance to P-glycoprotein-mediated resistance and the presence of experimental or preclinical evidence. Only studies meeting the inclusion criteria were retained for qualitative synthesis.

#### 2.5 Data extraction and analysis

Relevant information was extracted from selected studies, including nanoparticle type, mechanism of action against P-glycoprotein, experimental models (cell lines or animal models), type of drug delivered, and therapeutic outcomes. Special attention was given to multifunctional systems incorporating targeting ligands, stimuli-responsive release mechanisms, and co-delivery strategies such as siRNA or natural P-glycoprotein inhibitors. This approach enabled identification and comparative assessment of the most promising nanocarrier systems designed to overcome MDR.

#### 2.6 Quality considerations and limitations

Because this work is a narrative rather than systematic review, no formal risk-of-bias assessment or quantitative meta-analysis was conducted. Instead, emphasis was placed on selecting peer-reviewed studies with robust experimental design and clear mechanistic insight. However, several limitations should be acknowledged: (i) a potential selection bias inherent to the narrative design, (ii) a heterogeneity of experimental models, including differences in nanoparticle types, biological systems, and evaluation methods, and (iii) a lack of standardized reporting metrics (e.g., IC_50_ values, dosing regimens, and MDR assessment criteria), which limits direct comparison across studies. Despite these limitations, the adopted methodology enables a comprehensive and mechanism-oriented synthesis of emerging nanocarrier strategies targeting P-glycoprotein-mediated drug resistance.

### 3 Nanotechnology strategies against multidrug resistance

Multidrug resistance represents a major challenge in cancer therapy, often resulting from the overexpression of efflux transporters such as P-gp, MRP1, MRP2, and BCRP. ATP-binding cassette transporters actively expel chemotherapeutic drugs from cancer cells, resulting in diminished intracellular drug concentrations and impaired treatment effectiveness [26]. Nanotechnology-based drug delivery systems have emerged as a promising strategy to overcome this barrier. These nanosystems can encapsulate anticancer agents, protect them from premature degradation, and release them in a controlled and targeted manner. Furthermore, numerous nanocarriers are specifically engineered to inhibit or bypass efflux mechanisms facilitated by P-gp and other transporters, thereby enhancing intracellular drug accumulation and cytotoxic efficacy in tumor cells.

Nanocarriers may exhibit dual functionality by simultaneously delivering therapeutic agents while some excipients may also modulate P-glycoprotein activity via their components [27]. By utilizing the distinctive pathophysiological traits of tumors, such as the enhanced permeability and retention (EPR) effect, these biocompatible systems may improve drug pharmacokinetics and pharmacodynamics. Encapsulation within nanoparticles minimizes off-target toxicity, prolongs systemic circulation, and increases the therapeutic index [28]. A wide variety of nanocarriers have been explored for this purpose, including polymeric micelles, metallic nanoparticles, liposomes, exosomes and extracellular vesicles, functionalized liposomes, and mesoporous silica nanoparticles. Each platform offers distinct advantages depending on the drug, tumor type, and specific resistance mechanisms targeted.

#### 3.1 General classification by mechanism of action

**Figure 2:**
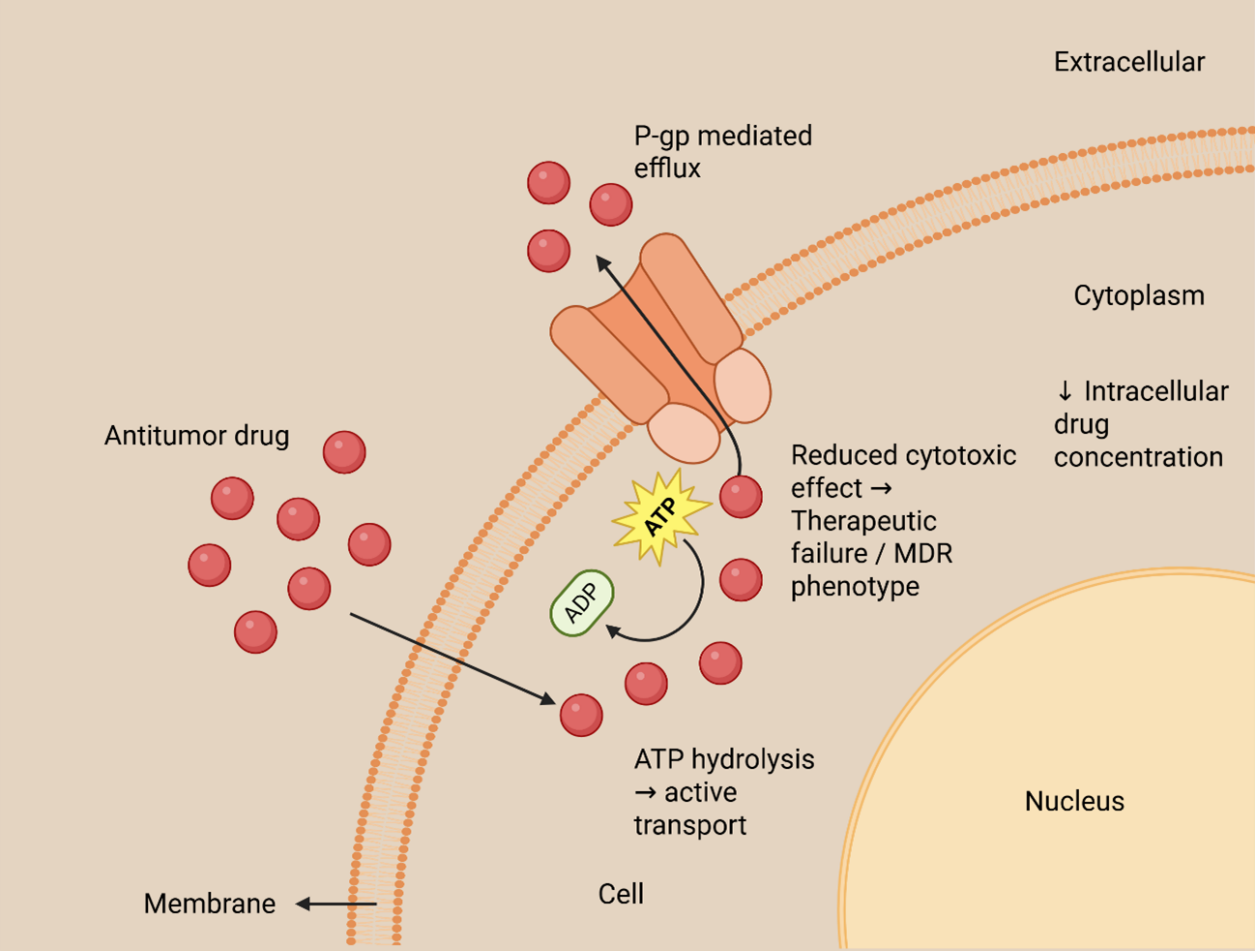
P-glycoprotein-mediated drug efflux mechanism. Overexpression of P-gp (ABCB1) in resistant cancer cells actively transports chemotherapeutic drugs out of the cytoplasm using ATP hydrolysis, thereby reducing intracellular drug concentration and cytotoxic efficacy. This energy-dependent efflux represents a key mechanism underlying MDR.

**3.1.1 Direct inhibition of P-gp.** (i) The ATPase function of P-gp can be directly inhibited through compounds such as quercetin, ᴅ-α-tocopheryl polyethylene glycol succinate (TPGS), celecoxib, imatinib, or tariquidar, encapsulated in nanoparticle systems. These agents may interact with substrate-binding regions of P-gp or interfere with ATPase-driven transport activity to block drug efflux, increasing intracellular concentration and drug efficacy. Negi et al. developed liposomes coated with hyaluronic acid and containing imatinib mesylate, a substrate of the P-gp and BCRP transporters (HA-LIPO-IM), to target CD-44- and P-gp-overexpressing colon cancer cells (HT-29 and Colo-320). The interaction of the liposomal particles showed the ability to modulate the action of P-gp by inhibiting ATPase activity, thanks to the excipients that constituted them, thereby increasing intracellular accumulation of the encapsulated drug with reduced influence of P-gp-mediated efflux [29]. As shown in Figure 2, direct inhibition of P-gp ATPase activity occurs when nanoparticles encapsulate inhibitors such as quercetin, TPGS, or tariquidar, which bind to the ATP-binding domains of P-gp, thereby preventing ATP hydrolysis and drug efflux. This results in higher intracellular drug accumulation and enhanced cytotoxicity. (ii) The co-administration of standard antineoplastic drugs with nanoparticle-encapsulated P-gp inhibitors, a combined use of drugs and MDR inhibitors, has demonstrated synergistic effects on MDR reversal. It increases therapeutic efficacy and intracellular drug concentrations while at the same time reducing systemic toxicity and premature drug degradation. For example, aiming to overcome the resistance developed by erlotinib (Ertb) in non-small cell lung cancer, Ganthala et al. prepared solid lipid nanoparticles (EQNPs) loaded with erlotinib, an anticancer drug, and quercetin (Quer), a natural product that increases apoptosis and autophagy in cancer. The authors found that this combination generated a synergistic effect against A549 and NCI H460 cells, overcoming the limitations of free therapies. EQNPs increased Ertb and Quer uptake while decreasing P-gp and nuclear epidermal growth factor receptor expression [30].

**Figure 3:**
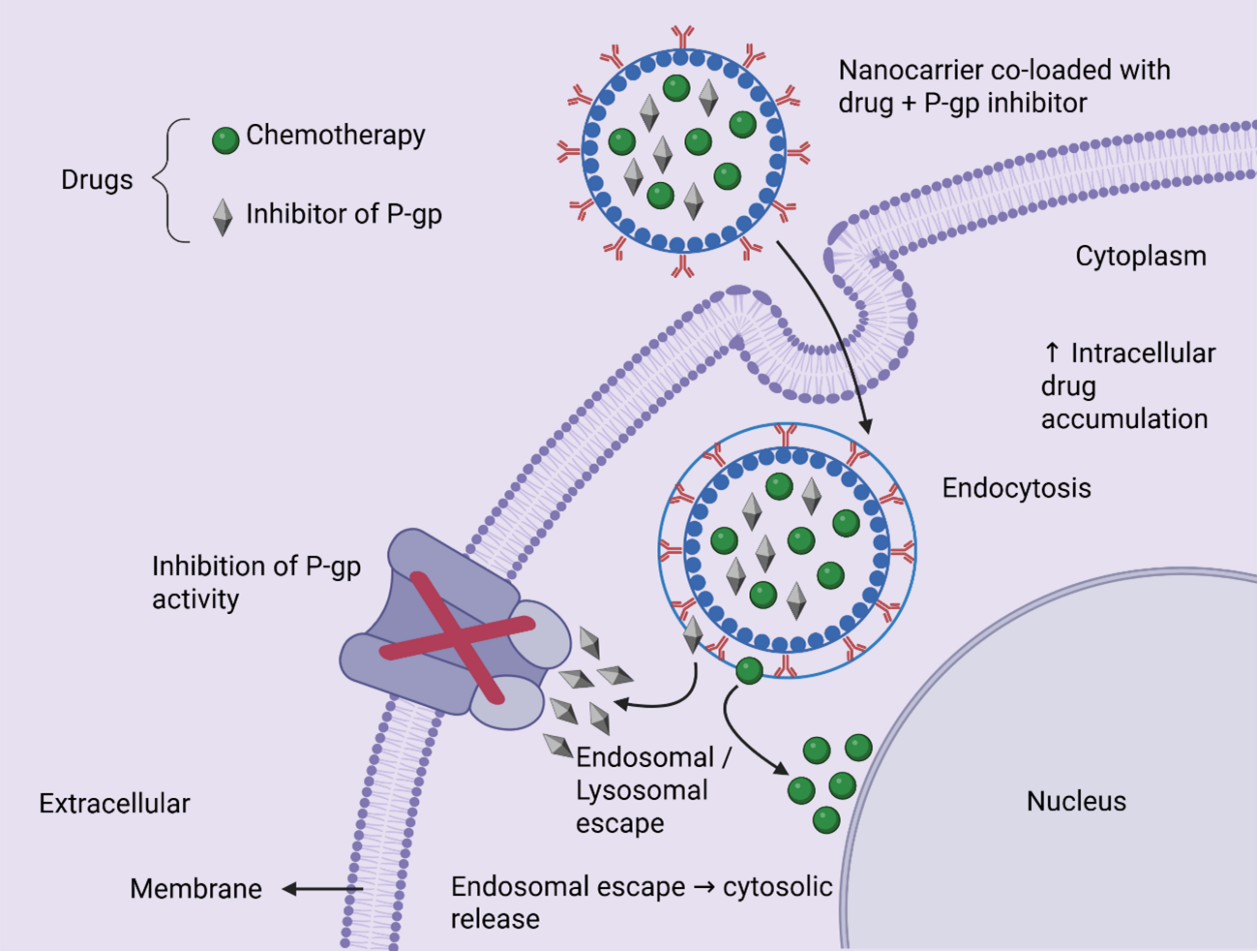
Nanocarrier co-delivery of chemotherapeutic agents and P-gp inhibitors. Nanoparticles co-loaded with a cytotoxic drug and a P-gp inhibitor (e.g., quercetin, TPGS, verapamil, or tariquidar) enter the cell through endocytosis and release their payload after endosomal escape. The inhibitor suppresses P-gp ATPase activity while the drug accumulates in the cytoplasm, leading to enhanced intracellular concentration and restoration of chemosensitivity.

Another study aimed at increasing the efficiency of two chemotherapeutic drugs and reducing their adverse effects combined doxorubicin (DOX) and chloroquine (CQ) in individual nanoparticles (NP_DOX+CQ_). In A549 and A549/Taxol cells, this co-delivery strategy enabled chloroquine to inhibit autophagy and protect doxorubicin from degradation, thereby increasing its accumulation in cancer cells. The fact that the drugs are encapsulated in NP_DOX+CQ_ prevents them from being recognized and expelled by P-gp [31]. Figure 3 shows the nanocarrier-based co-delivery of chemotherapeutic agents and P-gp inhibitors. This combined mechanism promotes endocytosis-mediated uptake, endosomal escape, and intracellular release, synergistically inhibiting P-gp activity and increasing the concentration of cytotoxic drugs in resistant tumor cells.

**Figure 4:**
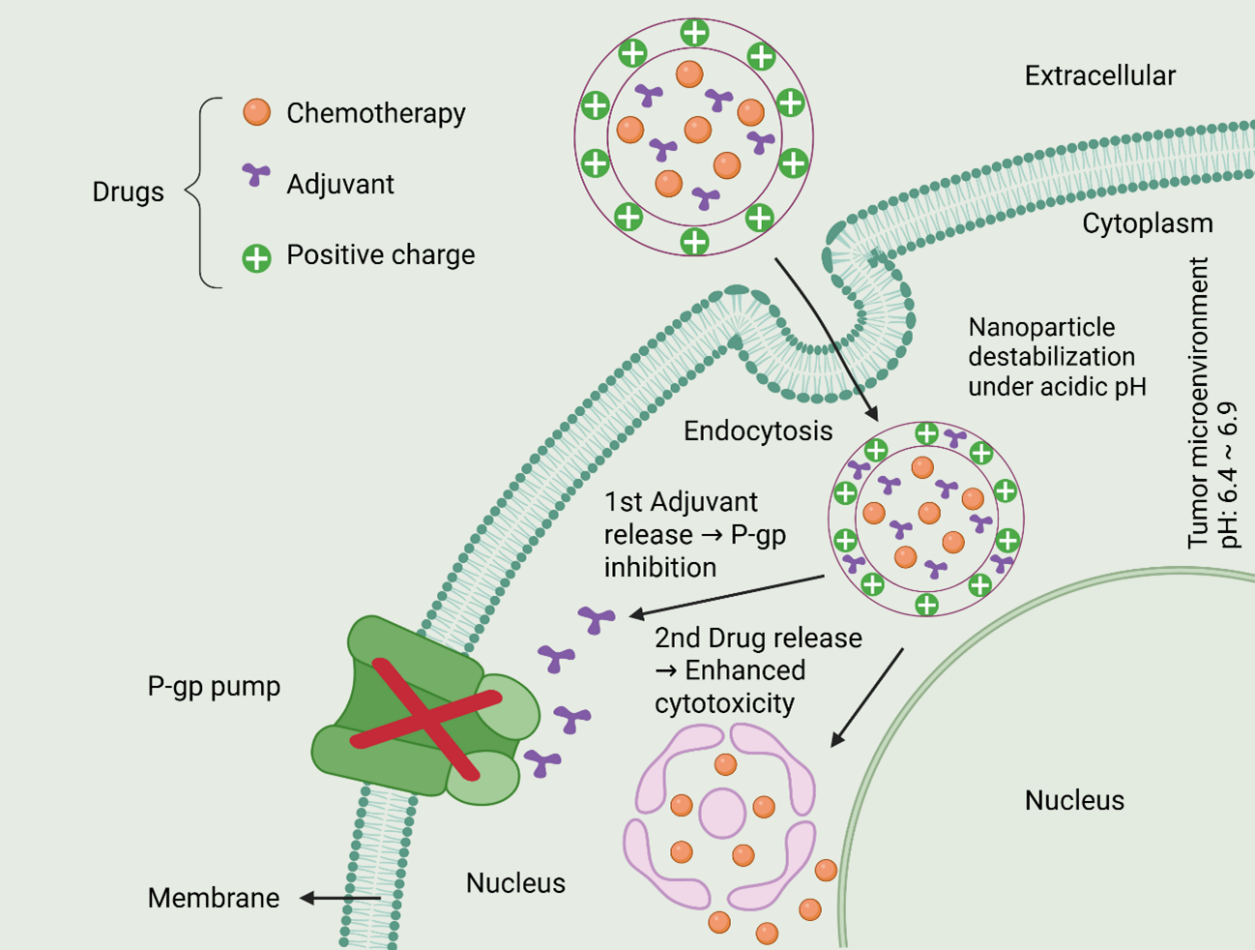
pH-responsive dual-stage drug release in the tumor microenvironment. pH-sensitive nanoparticles remain stable in circulation but destabilize under the mildly acidic conditions of tumor tissue (pH 6.4–6.9). This triggers a first-stage release of adjuvants that inhibit P-gp activity, followed by a second-stage release of the chemotherapeutic drug. The sequential release enhances intracellular drug retention and therapeutic efficacy.

**3.1.2 Strategies to evade P-gp-mediated efflux.** Advanced nanoparticle drug delivery systems exploit alternative cellular entry pathways, such as vesicular transport, organelle targeting, or specific features of the tumor microenvironment (e.g., acidic pH or elevated levels of reactive oxygen species (ROS)), to gain access to tumor cells. By entering through these routes, they may initially evade recognition by membrane-associated P-gp. However, once the drug is released into the cytosol, subsequent prevention of drug efflux remains essential. To achieve this, such systems must incorporate additional design features that promote sustained intracellular retention, ensuring the therapeutic agent remains within the cell long enough to exert its full effect. (i) Regarding receptor-mediated endocytosis, An et al. designed ApoA1-modified cationic liposomes loaded with doxorubicin (ApoA1-lip/Dox) to overcome MDR generated by overexpression of the P-gp efflux pump in the MCF-7/ADR breast cancer line. They showed that ApoA1-lip/Dox promotes greater cellular uptake of doxorubicin and generates fewer adverse effects, thanks to SR-B1 (scavenger receptor, class B type 1)-mediated endocytosis as a form of cellular internalization [32]. (ii) To achieve a release in response to the tumor microenvironment, nanoparticles are designed to be sensitive to tumor-specific stimuli, such as acidic pH or ROS so that the nanoparticles remain stable in the circulation and only release their cargo into the tumor tissue. Chen et al. designed liposomes with folate linkers co-loaded with doxorubicin and imatinib (FPL-DOX/IM) that remained stable during their passage through the blood circulation, but exhibited rapid drug release once they reached the acidic microenvironment of the tumor. The two key factors in this design are that the liposomes are pH-sensitive and that, once they fuse with the tumor membrane, imatinib is responsible for regulating the efflux levels of P-gp and BCRP transporters, enhancing the sensitivity of tumor cells to DOX [33]. As shown in Figure 4, a pH-responsive, dual-stage release system destabilizes the acidic TME (pH 6.4–6.9). The first release involves adjuvants that inhibit P-gp, followed by the release of the chemotherapeutic drug, thereby enhancing intracellular retention and therapeutic efficacy.

**Figure 5:**
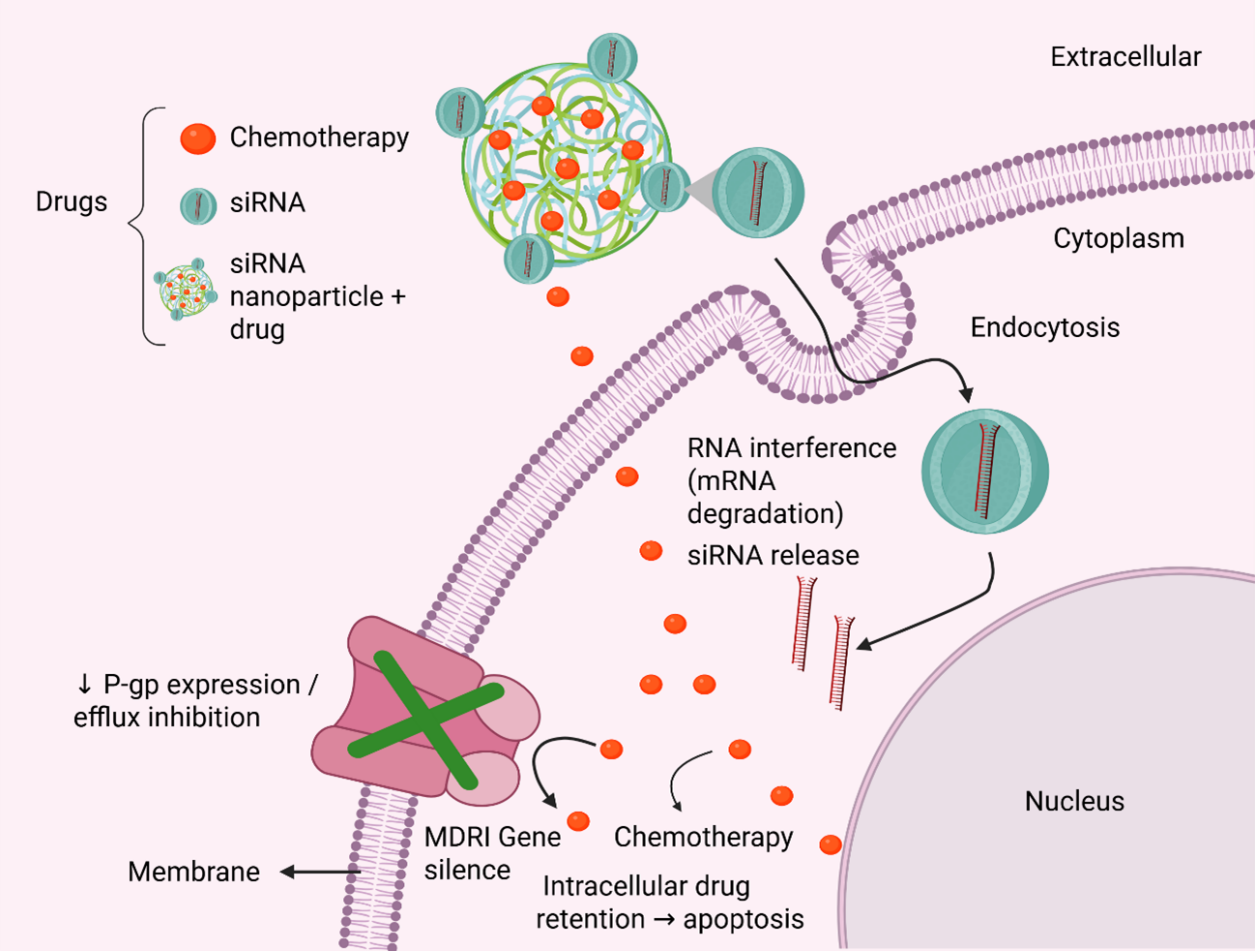
Gene-silencing nanocarriers co-loaded with siRNA and chemotherapeutic drugs. Nanocarriers deliver siRNA molecules that mediate RNA interference, degrading multidrug-resistance gene 1 (MDR1) mRNA and reducing P-gp synthesis. Genetic modulation decreases drug efflux and increases intracellular drug retention, while the co-delivered chemotherapeutic agent induces apoptosis in resistant tumor cells.

**3.1.3 Genetic modulation and expression of P-gp.** Nanoparticles can be engineered to deliver gene-silencing agents directly to resistant tumor cells, thereby suppressing P-gp expression at transcriptional or post-transcriptional levels, through epigenetic modifications, and restoring drug sensitivity. (i) The design of stable nanoscale assemblies co-loaded with siRNA to silence the expression of the ABCB1 (MDR1) gene, which encodes P-gp, is a promising option for overcoming MDR. Whang et al. designed nanosystems coated with hyaluronic acid (HA) and co-administered paclitaxel (PTX)/siRNA, aiming to inhibit P-gp levels and block drug efflux in taxol (A549/T)-resistant lung cells [34]. As shown in Figure 5, gene-silencing nanocarriers co-loaded with siRNA and chemotherapeutic agents operate through a gene-silencing mechanism. Following endocytosis, siRNA is released into the cytoplasm, where it mediates RNA interference and promotes MDR1 mRNA degradation. This suppresses P-gp synthesis, reduces efflux capacity, and restores drug sensitivity in multidrug-resistant tumor cells. (ii) Regarding epigenetic or miRNA modulation to reduce P-gp synthesis and expression, microRNA-155 (miR-155) is a class of endogenous non-coding RNAs of 18–25 nucleotides, which are associated with cancer development, progression, and metastasis. Aiming to resensitize colorectal cancer tumors to 5-fluorouracil (5-FU), Li et al. conducted a combined treatment trial of 5-FU and mesoporous silica nanoparticles (MSNs) loaded with anti-miR-155 (an oncogene) (MSNs-anti-miR-155@PDA-Apt). They observed that it blocked miR-155 expression, increased 5-FU cytotoxicity, and down-regulated P-gp [35].

Although siRNA- and miRNA-based strategies offer remarkable potential for reversing multidrug resistance, their therapeutic specificity requires careful control. P-gp plays essential physiological roles in normal tissues, such as blood–brain barrier, liver, and kidneys, where it contributes to detoxification and xenobiotic efflux. Consequently, non-selective silencing could lead to adverse effects such as altered pharmacokinetics or increased neurotoxicity.

To mitigate these risks, recent studies employ tumor-targeting ligands such as hyaluronic acid, folate, transferrin, and others that can selectively recognize overexpressed receptors on resistant cancer cells, as well as stimuli-responsive nanocarriers that release siRNA under acidic or enzymatic conditions typical of the TME. These integrated designs not only improve the safety profile of gene-silencing therapies but also reinforce the synergistic behavior between chemotherapeutic agents and genetic payloads, resulting in enhanced intracellular accumulation, restoration of drug sensitivity, and reduced off-target effects. Such multifunctional nanoplatforms represent the growing shift from single-mechanism to precision-targeted therapeutic systems within the field of P-gp modulation.

**3.1.4 Generation of oxidative stress or mitochondrial dysfunction.** Unlike direct inhibition of drug efflux pumps, this strategy focuses on disrupting cellular homeostasis, thereby promoting apoptosis and, in some cases, increasing intracellular drug retention in resistant cancer cells. A clear example is nanoparticles loaded with ROS-inducing compounds such as celecoxib (CEL). CEL at low concentrations and co-administered with DOX in liposomes has been shown to disrupt cellular respiration and induce ROS production directly from the mitochondria, leading to down-regulation of P-glycoprotein overexpression and, consequently, to increased retention and intracellular accumulation of DOX in MCF/ADR cells [36].

Structural analyses, drug combinations with synergistic effects, the use of natural extracts, and the study of potent, non-toxic, and specific inhibitors against P-gp have been extensively reported in other reviews. In this review, we will focus on the mechanism of drug efflux mediated by P-glycoprotein (P-gp or MDR1/ABCB1) overexpression and the use of different types of nanoparticles to prevent and inhibit P-gp efflux to overcome MDR.

**3.1.5 Comparative analysis of nanotransporter strategies.** Among the nanoparticle-based strategies to overcome P-gp-mediated MDR, direct inhibition may provide the most immediate functional reversal of efflux in tumors with moderate P-gp overexpression (e.g., breast, ovarian, and colon cancers) as the co-delivery of inhibitors like tariquidar or elacridar with chemotherapeutics can restore drug accumulation efficiently; however, its clinical potential remains limited by systemic toxicity and transporter-related pharmacokinetic alterations [37–38]. Efflux evasion through endocytotic uptake and stimuli-responsive release is highly versatile and particularly suited to solid tumors with poor vascularization and acidic microenvironments (e.g., pancreatic cancer or glioblastoma), where bypassing the plasma membrane efflux pump is advantageous; it shows good translational promise due to biocompatibility and tunable release control [33]. Gene silencing of ABCB1 via siRNA or CRISPR-loaded nanoparticles is the mechanistically most specific approach and ideal for tumors with high stable P-gp expression (e.g., leukemias and drug-refractory breast cancers), but clinical translation is hampered by nucleic acid instability and delivery barriers [34,39]. Finally, oxidative stress-based strategies are broadly effective as ROS overproduction can suppress P-gp and induce apoptosis regardless of efflux levels, making them attractive for redox-dysregulated tumors (e.g., prostate cancer and hepatocellular carcinoma); however, their narrow therapeutic window poses safety challenges [40–42]. Overall, efflux evasion currently appears to hold the greatest translational potential due to its generality, safety, and adaptability to tumor microenvironmental cues, while gene silencing remains the most promising for next-generation precision MDR therapy once delivery hurdles are overcome (Table 1).

**Table 1:** Comparison of nanoparticle-based strategies to overcome P-gp-mediated multidrug resistance.

	Direct inhibition	Efflux evasion	Genetic modulation	Targeting oxidative/mitochondrial metabolism

primary target	P-gp protein (functional inhibition)	cellular entry pathway	ABCB1 gene expression	cellular redox balance and energy metabolism
temporal scale of effect	rapid, reversible	rapid, reversible	slower, long-lasting	rapid, potentially irreversible
sustainability of MDR reversal	temporary (requires continuous inhibition)	temporary (depends on nanoparticle presence)	long-term effect (gene silencing)	long-term (induces apoptosis)
specificity	moderate (possible off-target inhibition)	high (depending on targeting ligands)	high (sequence-specific)	low to moderate (may affect normal cells)
toxicity risk	moderate (systemic inhibitory effects)	low (depends on nanocarrier biocompatibility)	low to moderate (vector-related safety)	high (ROS-induced collateral damage)
formulation complexity	moderate (co-loading of drug + inhibitor)	low to moderate	high (requires nucleic acid stabilization)	variable (depends on nanoparticle composition)
potential synergy with chemotherapy	strong (directly enhances drug retention)	strong (increases intracellular drug accumulation)	very strong (reduces basal P-gp levels)	moderate to strong (induces efflux-independent apoptosis)

#### 3.2 Classification by type of nanoparticles

**3.2.1 Lipid-based nanoparticles.** Lipid nanoparticles (LNPs) are nanoscale drug delivery systems composed of lipids that enable the encapsulation and transport of active substances such as drugs, nucleic acids, proteins, and other therapeutic compounds [43]. These structures reduce systemic toxicity and improve therapeutic efficacy and enhance therapeutic efficacy by improving solubility, stability, bioavailability, and controlled drug release. Additionally, their low toxicity, scalability, and manufacturing feasibility make them attractive for both clinical and industrial applications [44]. There are several types of LNPs, including liposomes, first discovered in the 1960s, which were commonly used to deliver anticancer agents, antibiotics, and vaccines. The size, surface charge, and composition of liposomes influence their biological behavior, biodistribution, and shelf life [45].

Another important class are nanostructured lipid carriers (NLCs), which incorporate liquid lipids into a solid lipid matrix. This design prevents excessive crystallization, enhances stress resistance, and allows for more efficient and controlled release of active compounds. NLCs are typically categorized into amorphous, imperfect, and multitype structures [46]. Examples of NLCs are presented in Table 2.

**Table 2:** Lipid-based nanoparticle strategies against multidrug resistance.^a^

Nanoparticle used	1: Encapsulated drug; 2: Mechanism of action; 3: Experimental results; 4: Model; 5: MDR criterion; 6: Primary outcome measure	Ref.

Type of cancer: breast cancer		

solid lipid nanoparticles coated with chitosan	1: curcumin (CUR) 2: improved CUR delivery, inhibited P-gp, and reversed doxorubicin resistance 3: chitosan coating had no added benefits; reduced P-gp expression without systemic or cardiac toxicity; increased tumor doxorubicin accumulation 4: in vitro + in vivo (MCF-7/ADR; murine xenograft) 5: DOX resistance (ABCB1 overexpression) 6: P-gp expression (WB); intratumoral DOX accumulation	[47]
functional liposomes modified with hybrid peptide MTS-R8H3	1: doxorubicin (DOX) + celecoxib (CEL) 2: lysosomal escape, mitochondrial accumulation, ROS induction, and P-gp suppression 3: increased cytotoxicity; 5.72× higher cellular uptake; 18.56× more ROS production than control 4: in vitro (MCF-7/ADR) 5: DOX resistance (P-gp overexpression) 6: cellular uptake; ROS; cytotoxicity (IC_50_)	[36]
ApoA1-modified liposomes	1: doxorubicin (DOX) 2: SR-B1/clathrin-mediated endocytosis; reduces DOX efflux by P-gp; induces apoptosis (↓Bcl-2, ↑caspase-3). 3: higher intracellular DOX than unmodified liposomes; tumor growth inhibition in vivo; reduced cardiotoxicity 4: in vitro + in vivo (MCF-7/ADR) 5: DOX efflux via P-gp 6: intracellular DOX; tumor volume; cardiac toxicity	[32]
lipid nanoparticles Qu-SS-Gcc (glycerol caprylate-caprate modified with quercetin and disulfide linkages)	1: paclitaxel (PTX) 2: disulfide cleavage releases PTX intratumorally; quercetin inhibits P-gp, reducing efflux 3: rapid quercetin/PTX release; reduced toxicity on normal cells; substantial tumor accumulation and decreased tumor volume in vivo 4: in vitro + in vivo 5: P-gp-mediated PTX efflux 6: tumor volume; PTX retention	[48]
folate-modified pH-sensitive liposomes (FPL-DOX/IM)	1: doxorubicin (DOX) + imatinib (IM) 2: pH-sensitive release; folate targeting; IM inhibits ABC transporters (P-gp, BCRP), reducing DOX efflux 3: >90% DOX/IM release in 72 h at acidic pH; enhanced antitumor efficacy; reduced organ toxicity 4: in vitro + in vivo 5: ABCB1/ABCG2 overexpression 6: DOX release; antitumor efficacy; organ toxicity	[33]
liposomes modified with TPGS (ᴅ-α-tocopheryl PEG 1000 succinate; P-gp inhibitor)	1: paclitaxel (PTX) 2: TPGS inhibits P-gp; improved PTX release; reduced side effects; enhanced uptake and cytotoxicity 3: sustained PTX release over 72 h; 3.56× and 5.75× higher uptake at 2 h/4 h in MCF-7/ADR; greater cytotoxicity; inhibited P-gp expression 4: in vitro (MCF-7/ADR) 5: P-gp overexpression (Rh123 efflux) 6: P-gp expression; PTX uptake; IC_50_	[49]
self-assembled mixed phospholipid nano-micelles	1: mangiferin (Mgf) 2: TPGS and PEG inhibit P-gp; improved cellular accumulation and intestinal permeability 3: NPs <60 nm, >80% release in 15 min; higher cytotoxicity and accumulation in MCF-7; 6.6× higher bioavailability in rats; no toxicity in vital organs 4: in vitro + in vivo (rats) 5: intestinal P-gp efflux 6: IC_50_ (MCF-7); bioavailability	[50]
TPGS-coated liposomes	1: docetaxel (DTX) 2: P-gp inhibition improves intracellular DTX accumulation and helps overcome MDR. 3: TPGS liposomes (≈140 nm) enhanced DTX cytotoxicity and accumulation in MCF-7 and MCF-7/ADR cells; apoptosis via synergy 4: in vitro (MCF-7; MCF-7/ADR) 5: P-gp-mediated DTX efflux 6: intracellular DTX; apoptosis	[51]
PEGylated liposomes co-encapsulate disulfiram (DSF, bilayer) and doxorubicin (DOX, core)	1: disulfiram (DSF) + doxorubicin (DOX) 2: DSF-induced P-gp sulfhydration/ubiquitination; enhanced DOX retention; distinct release kinetics 3: enhanced DOX retention/cytotoxicity in P-gp+ breast cancer cells; improved tumor suppression; reduced systemic toxicity 4: in vitro + in vivo 5: P-gp overexpression 6: P-gp expression (WB); DOX retention	[52]
solid lipid nanoparticles	1: paclitaxel (PTX) 2: bypassed P-gp efflux via caveola-mediated endocytosis; increased cytotoxicity in MDR cells 3: enhanced PTX uptake and cytotoxicity in MCF-7/ADR; caveola-mediated uptake in MDR vs clathrin-independent in sensitive cells 4: in vitro (MCF-7/ADR) 5: P-gp-mediated PTX efflux 6: PTX uptake; cytotoxicity	[53]
PEGylated liposomes	1: doxorubicin (DOX) + quercetin (QUE) 2: quercetin inhibits P-gp; PEGylation improves circulation and tumor localization (EPR); liposome improves absorption and reduces toxicity 3: 4.81× and 3.21× cytotoxicity enhancement in HL-60/ADR and MCF-7/ADR; enhanced tumor suppression; reduced cardiac toxicity 4: in vitro + in vivo 5: P-gp-mediated DOX efflux 6: cytotoxicity enhancement; cardiac toxicity	[54]
hybrid liposome–mesoporous silica nanoparticles (HLM-N@DOX/R) – lipid-coated inorganic core	1: doxorubicin (DOX) + P-gp siRNA 2: pH/redox-responsive release; siRNA reduces P-gp; liposomal coating improves biocompatibility and uptake; BAX-mediated apoptosis 3: reduced P-gp by 80%; 71% tumor suppression in MDR xenografts; efficient tumor inhibition; reduced systemic toxicity 4: in vitro + in vivo (MDR breast xenograft) 5: P-gp expression (WB) + ABCB1 silencing 6: P-gp expression; tumor suppression (%)	[55]
redox-responsive mesoporous silica nanoparticles coated with lipid bilayers (HT-LMSNs-SS)	1: doxorubicin (DOX) 2: disulfide redox-release; TPGS inhibits P-gp ATPase; HA enables CD44 targeting; ROS apoptosis 3: increased DOX retention in MCF-7/ADR; significant tumor inhibition in MDR xenograft; P-gp suppression; minimal systemic toxicity 4: in vitro + in vivo 5: P-gp ATPase inhibition; ROS 6: P-gp expression; tumor inhibition	[56]
T7 peptide-modified mixed liposomes (T7-MLP@DTX/SchB)	1: docetaxel (DTX) + schisandrin B (SchB) 2: T7 peptide targets TfR; SchB inhibits P-gp; co-delivery enhances synergy; reduced systemic toxicity 3: increased DTX accumulation in MDR MCF-7/DR; significant tumor inhibition; enhanced chemosensitivity; improved biocompatibility 4: in vitro + in vivo 5: P-gp-mediated DTX efflux 6: DTX accumulation; tumor inhibition	[57]
lipid-coated polymeric metformin (PolyMet)/poly-γ-glutamic acid-DOX nanoparticles (LPPD) – lipid-polymer hybrid	1: doxorubicin (DOX) + PolyMet 2: PolyMet inhibits ABCB1/ABCC1; AMPK/mTOR activation sensitizes tumor cells; lipid coating improves stability and accumulation; pH-responsive release 3: enhanced DOX accumulation in MCF-7/DOX; notable tumor growth suppression with less systemic toxicity; ABC transporter inhibition 4: in vitro + in vivo 5: ABCB1 + ABCC1 inhibition 6: DOX accumulation; tumor volume	[58]
dual drug-loaded LIPID and LIPID-HYBRID nanocarriers (liposomes, SLNs, and lipid–polymer hybrid NPs)	1: doxorubicin (DOX) + chemosensitizer (curcumin, metformin, resveratrol, elacridar, mitomycin-C, disulfiram, or docosahexaenoic acid) 2: co-delivery of DOX + chemosensitizer increases intracellular accumulation; P-gp efflux inhibition overcomes MDR; synergistic cytotoxicity and apoptosis 3: enhanced DOX retention and cytotoxicity in MDR breast cancer cells; reduced systemic toxicity; efficacious tumor suppression in multiple in vitro/in vivo MDR models 4: in vitro + in vivo (multiple MDR models) 5: P-gp-mediated DOX efflux 6: DOX retention; tumor suppression	[59]

Type of cancer: non-small cell lung cancer		

solid lipid nanoparticles	1: erlotinib (Ertb) + quercetin (Quer) 2: co-delivery to improve solubility and permeability 3: drug-loaded EQNPs exhibited synergistic action; inhibited P-gp and nuclear EGFR in A549/ER cells; improved bioavailability and therapeutic effect 4: in vitro (A549/ER) 5: P-gp + nuclear EGFR 6: P-gp expression; bioavailability; cytotoxicity	[30]

^a^Abbreviations: SLNs: Solid lipid nanoparticles; DOX: doxorubicin; CUR: curcumin; CEL: celecoxib; PTX: paclitaxel; DTX: docetaxel; IM: imatinib; DSF: disulfiram; QUE/Qu: quercetin; SchB: schisandrin B; TPGS: ᴅ-α-tocopheryl polyethylene glycol 1000 succinate; Mgf: mangiferin; Ertb: erlotinib; TfR: transferrin receptor; HA: hyaluronic acid; EPR: enhanced permeability and retention; MDR: multidrug resistance; P-gp: P-glycoprotein (ABCB1); MCF-7/ADR: doxorubicin-resistant MCF-7; ROS: reactive oxygen species; WB: western blot; SR-B1: scavenger receptor class B type 1.

**3.2.1.1 Functionalized liposomes.** Surface functionalization of liposomes aims to deliver anticancer agents directly to tumors by attaching specific ligands, such as peptides, antibodies or their fragments, or small molecules. This strategy leverages the overexpression of specific receptors on cancer cells, significantly enhancing therapeutic efficacy while minimizing side effects on healthy tissues [60]. There are various types of liposomes designed according to their therapeutic applications. Multifunctional liposomes, for instance, can combine different ligands or multiple drugs to integrate both therapeutic and diagnostic functions [61].

Functionalized liposomes demonstrate the ability to overcome P-gp-mediated drug resistance through multiple mechanisms without relying on toxic first-generation inhibitors. A paradigmatic example are doxorubicin-loaded ApoA1 liposomes: Apolipoprotein A1 targets the liposomes to the SR-B1 receptor and triggers clathrin-mediated endocytosis, thereby increasing cellular uptake and apoptosis in MCF-7/ADR cells, while reducing cardiotoxicity and achieving 79% tumor inhibition in murine models [32]. With another strategy, the DOX/CEL-MTS-R8H3 system utilizes a hybrid peptide that facilitates cell penetration, lysosomal escape, and mitochondrial targeting. This leads to a 5.7-fold augmentation in intracellular drug absorption, improved ROS generation, and substantial suppression of P-gp efflux relative to non-peptide-functionalized liposomes [36].

Both methodologies affirm that the crux resides in activating internalization routes that circumvent the efflux pump while concurrently depleting its energy source or locally blocking it. Supporting this concept is the incorporation of TPGS into the liposomal bilayer. Acting as both a substrate and ATP inhibitor, TPGS immobilizes P-gp and triples docetaxel uptake in resistant cells, thereby increasing cytotoxicity [49–51]. Collectively, these findings suggest that the liposomal surface can be engineered as a “multitool” to combat MDR, that is, targeting via ApoA1, mitochondrial disruption via MTS-R8H3, and efflux inhibition via TPGS. The convergence of these strategies supports the development of modular nanocarriers that maximize intratumoral drug delivery while minimizing systemic toxicity, paving the way for future combinations with immunotherapies and tumor resensitization in clinical trials.

**3.2.1.2 Solid lipid nanoparticles.** Solid lipid nanoparticles (SLNs) are nanoscale structures composed of solid lipids and stabilizing agents. They provide superior biocompatibility and facilitate prolonged drug release [62]. Nonetheless, their utilization is constrained by a diminished ability to encapsulate hydrophobic substances and the possibility of structural changes with time. An exemplary case includes curcumin-loaded SLNs, which have shown effectiveness in counteracting P-gp-mediated resistance to doxorubicin in triple-negative breast cancer. These nanoparticles typically have diameters of less than 200 nm and an encapsulation efficiency of 70–75%. Upon cellular internalization, they significantly enhance intracellular retention of the anthracycline and increase its cytotoxicity five- to tenfold [63].

Chitosan coating, intended to improve hydrophilicity and colloidal stability, did not provide additional advantages compared to uncoated SLNs. Both formulations reduced ROS levels, inhibited the Akt/IKKα-β/NF-κB signaling pathway, and downregulated ABCB1 gene transcription, leading to decreased expression and activity of P-gp. In mouse models, the combination of curcumin-loaded SLNs with doxorubicin restored tumor sensitivity without inducing systemic or cardiac toxicity. These findings support curcumin-loaded SLNs, whether chitosan-coated or not, as biocompatible and safe nanocarriers for overcoming multidrug resistance associated with efflux transporter [47].

**3.2.1.3 Lipid–polymer hybrid nanoparticles.** Another important class of nanocarriers are lipid–polymer hybrid nanoparticles (LPNs), which integrate the advantageous properties of lipids (biocompatibility) and polymers (structural stability). These nanoparticles can exhibit various structural configurations, such as lipid shells surrounding polymeric cores or polycarbonates embedded in a uniform matrix. Due to their high encapsulation efficiency, LPNs are widely used for sustained drug release in cancer therapy and for applications in medical imaging [64].

LPNs typically combine a lipid bilayer or core with amphiphilic copolymers to enable, within a single system, active targeting, controlled drug release, and energetic inhibition of P-gp. A paradigmatic example is the HA-DOCA-His-PF micelle system: The hyaluronic acid–deoxycholic acid–histidine scaffold targets CD44 receptors on cancer cells. Upon endocytosis, the acidic endosomal pH destabilizes the micelle, triggering doxorubicin release. Concurrently, integrated Pluronic F127 diminishes ATP levels and obstructs P-glycoprotein function, facilitating drug buildup in the cytoplasm and subsequent nuclear translocation [65].

In MCF-7/ADR cells, these micelles markedly increased intracellular doxorubicin accumulation, decreased the resistance index from 45 to 1.9, and triggered apoptosis. In mouse xenograft studies, median life was extended to 43 days without any systemic toxicity. Beyond this design, the strategy has been replicated using hybrid systems containing TPGS or different types of Pluronic copolymers (e.g., F127/P123), where the polymeric segment modulates efflux activity, and the lipid portion contributes biocompatibility and a dense matrix for high drug loading [66].

In all cases, the result is a synergistic mechanism involving enhanced cellular uptake, stimuli-responsive drug release, and direct inhibition of P-gp activity. This collaboration results in substantial decreases in IC_50_ values and tumor inhibition rates over 80% in multidrug-resistant animals. The findings underscore the adaptability of lipid–polymer hybrid nanostructures as modular platforms for reengineering traditional anticancer medicines and enhancing their therapeutic effectiveness against resistant malignancies.

**3.2.2 Polymeric nanoparticles.** Polymeric nanoparticles are nanoscale pharmaceutical systems primarily composed of biocompatible and biodegradable polymers. These structures enable the encapsulation or conjugation of active compounds, allowing for controlled and targeted drug release [67]. One of their major advantages is the improvement of the bioavailability of poorly water-soluble or unstable drugs. They also protect sensitive molecules such as proteins and nucleic acids from premature degradation and rapid clearance by the body. Unlike other solid nanoparticles, such as metallic ones, polymeric nanoparticles can have empty or permeable internal structures that provide higher drug-loading capacity. Besides, their surface can be functionalized with polymers like polyethylene glycol (PEG) and particular ligands, improving their circulation, minimizing resistant framework acknowledgement, and promoting targeted delivery to particular tissues [68]. These nanoparticles can be created utilizing either top-down strategies (from preformed polymers) or bottom-up methods (from monomers), with emulsification being one of the most commonly used techniques. Thanks to their chemical and structural versatility, they can be designed to reply to particular natural stimuli, such as changes in pH, temperature, or enzymatic activity, and discharge their payload only under desired conditions [69]. These properties have made polymeric nanoparticles exceedingly promising for different applications, from delivery of anticancer drugs to the advancement of biosensors, as well as in the nutrition industry [70]. Whereas clinically approved formulations such as Eligard^®^ (a PLGA-based in situ forming implant), and Genexol-PM^®^ (a PEG–PLA polymeric micelle for cancer treatment) are already available, many current designs still face challenges, especially in understanding biological barriers, patient response variability, and the translation of preclinical findings into clinical practice [71]. Progress in design of polymers and multistage responsive frameworks opens ways for utilizing polymeric nanoparticles to create more viable, more secure, and personalized treatments. Examples of polymeric nanoparticles are presented in Table 3.

**Table 3:** Polymeric and polymer hybrid nanoparticles used against multidrug resistance.^a^

Type of cancer	Nanoparticles used	1: Encapsulated drug, 2: Mechanism of action, 3: Experimental results, 4: Model, 5: MDR criterion, 6: P-gp evidence type	Ref.

breast cancer	polymeric micelles – HA-DOCA-His-PF (hyaluronic acid–deoxycholic acid–histidine + Pluronic F127)	1: doxorubicin (DOX) 2: endosome-pH triggered DOX release + PF127-mediated P-gp efflux inhibition 3: ↑ cytotoxicity vs MCF-7/ADR; ↑ intracellular DOX; tumor growth inhibition in vivo 4: in vitro + in vivo (MCF-7/ADR) 5: P-gp efflux + resistance index 6: direct – intracellular DOX quantification (FACS) + RI measurement	[65]
breast cancer	polymeric NPs – γ-PGA-*g*-PLGA coated with cholesterol-PEG (C-PEG)	1: doxorubicin (DOX) 2: receptor-mediated endocytosis; P-gp inhibition retains DOX; photothermal release 3: enhanced uptake; synergy with PTT; favored accumulation in MDR xenografts 4: in vitro + in vivo (MDR tumor mice) 5: P-gp efflux 6: indirect – inferred from cytotoxicity + retention (requires orthogonal efflux assay)	[72]
breast cancer	polymeric micelles – TPGS-IDM (TPGS + indomethacin conjugate)	1: paclitaxel (PTX) 2: P-gp + MRP1 inhibition; ↓ ATP; ↑ ROS via mitochondrial damage → MDR reversal and apoptosis 3: ≈70% PTX release at pH 5.0/esterase; high internalization; 77.9% apoptosis in MCF-7/ADR; ROS↑, ATP↓ 4: in vitro (MCF-7/ADR) 5: P-gp + MRP1 + ATP 6: direct – ATP assay + apoptosis quantification + efflux assay	[73]
breast cancer	polymeric NPs – pH-sensitive poly(ortho ester urethane) (POEU)	1: doxorubicin (DOX) + pyrrolidine/diethyldithiocarbamate (PDTC) 2: PDTC chemosensitizer inhibits NF-κB, ↓ P-gp; pH-sensitive NPs release in acidic TME 3: enhanced DOX accumulation; 82.9% tumor inhibition in vivo; reduced cardiotoxicity 4: in vitro + in vivo (MCF-7/ADR xenograft) 5: NF-κB→P-gp axis 6: direct – P-gp WB + intracellular DOX accumulation	[74]
breast cancer	polymeric hybrid micelles – Pluronic F127 + P123-phenylboronic ester (PHE), H_2_O_2_-responsive	1: doxorubicin (DOX) 2: F127 stability/circulation; P123-PHE responsive to H_2_O_2_; inhibits P-gp/MRPs; ↓ GSH, ↑ ROS 3: F127/PHE-DOX superior cytotoxicity/apoptosis vs MCF-7/ADR; 87.87% tumor growth inhibition in vivo; ↓ cardiotoxicity 4: in vitro + in vivo 5: P-gp + MRPs + ROS/GSH balance 6: direct – efflux assay + ROS/GSH quantification	[66]
breast cancer	polymer–polymer HYBRID NP – PTX/PTS/HQ (PEI-TOS-SS + HA–quercetin)	1: paclitaxel (PTX) + quercetin (QU) 2: CD44 endocytosis; proton-sponge lysosomal escape; acidic pH releases QU → P-gp inhibition; PTX retention 3: IC_50_ 9.348 vs 44.377 µg/mL (free PTX) in MCF-7/ADR; ↑ apoptosis; 2.54× tumor volume reduction in vivo 4: in vitro + in vivo 5: P-gp-mediated PTX efflux 6: direct – IC_50_ + fluorescence biodistribution	[75]
breast cancer	polymeric mixed micelles – DSPE-PEG2000 + PEG-PCL	1: paclitaxel (PTX) + naringin (NRG) 2: naringin inhibits P-gp; ↑ PTX retention 3: 85.27%/53.55% release (PTX/NRG) in 4 h; 65% cytotoxicity at 15 µg/mL vs 50 µg/mL free PTX; ↑ uptake 4: in vitro (MCF-7) 5: P-gp-mediated PTX efflux 6: indirect – inferred from cytotoxicity + uptake (no direct P-gp functional assay reported)	[76]
breast cancer	polymeric micelles – pH-sensitive mPEG-PLA-PHis	1: doxorubicin (DOX) + resveratrol (Res) 2: pH-triggered release; Res inhibits P-gp, ↓ mitochondrial ATP, ↑ DOX cytotoxicity 3: pH-endoSM/DOX/Res reduced DOX IC_50_ 17.38× in MCF-7/ADR and showed excellent tumor accumulation in vivo 4: in vitro + in vivo (MCF-7/ADR) 5: P-gp + mitochondrial ATP 6: direct – IC_50_ fold-change + ATP assay	[77]
breast cancer	polymeric micelles – podophyllotoxin-polyacrylic acid (PPC) self-assembled	1: podophyllotoxin (PPT) 2: improved solubility; P-gp-independent cytotoxicity (PPT is not a P-gp substrate) 3: 215 ± 11 nm; CMC 0.430 mg/mL; ↑ solubility; ↓ hemolysis; ↑ cytotoxicity at 48 h 4: in vitro (MCF-7; MDA-MB-231) 5: non–P-gp substrate cytotoxicity 6: indirect – no P-gp functional assay (PPT is a non–P-gp substrate; claim is pharmacologically reasonable but unverified in this study)	[78]
breast cancer	polymeric micelles – cholecalciferol–PEG2000 conjugate (PEGCCF)	1: doxorubicin (DOX) 2: P-gp inhibition; ↓ mTOR/c-Myc/Bcl-xL; ↑ Bax → apoptosis. 3: 40 ± 8.7 nm; PEGCCF-DOX ↑ DOX cytotoxicity (1.8–2.9×); ↓ P-gp activity; ↑ apoptosis (10× in MDA-MB-231DR) 4: in vitro (MDA-MB-231/DR) 5: P-gp activity + apoptosis pathway 6: direct – P-gp activity assay reported	[79]
breast cancer	polymer–drug conjugate micelles – TPGS2000-DOX prodrug	1: doxorubicin (DOX) 2: passive EPR; acid-sensitive DOX release; TPGS-mediated P-gp reversal 3: ↑ tumor accumulation and uptake; higher cytotoxicity vs MCF-7/ADR; improved tumor suppression in vivo 4: in vitro + in vivo 5: P-gp-mediated DOX efflux 6: indirect – inferred from retention and cytotoxicity	[80]
breast cancer	polymeric micelles – ROS-responsive protoporphyrin IX (PpIX)-conjugated	1: apatinib + doxorubicin (DOX) 2: PpIX-mediated PDT generates ROS; ROS triggers micelle disassembly; apatinib inhibits P-gp 3: ↑ DOX accumulation/retention in MDR cells; significant tumor inhibition; effective P-gp suppression; synergistic chemo-PDT 4: in vitro + in vivo 5: P-gp + ROS/PDT 6: direct – P-gp WB + DOX accumulation	[81]
breast cancer	polymeric nanovesicle – pH-responsive mPEG-P[Asp(HPA-g-DOX)-BLA]	1: doxorubicin (DOX) + elacridar (Ela) 2: acid-cleavable amide release; elacridar inhibits P-gp 3: ↑ DOX retention in MCF-7/ADR; greater cytotoxicity; tumor suppression in vivo 4: in vitro + in vivo 5: P-gp-mediated DOX efflux 6: direct – Elacridar is a validated P-gp inhibitor; efflux confirmed	[82]
breast cancer	polymer–drug prodrug NP – reduction/photo dual-responsive (RPDRD)	1: doxorubicin (DOX) + P-gp siRNA 2: GSH-triggered siRNA release; suppresses P-gp expression; light-triggered DOX release 3: ↑ DOX retention in MCF-7/ADR; ↑ cytotoxicity; significant tumor inhibition in vivo 4: in vitro + in vivo 5: P-gp silencing via siRNA 6: direct – siRNA-mediated P-gp knockdown verified	[83]
breast cancer	polymeric micelles – pH-sensitive star-shaped TPGS copolymer	1: doxorubicin (DOX) 2: pH-responsive disassembly; TPGS-mediated P-gp inhibition; DOX/TPGS synergy 3: ↑ DOX accumulation/retention in MCF-7/ADR; significant tumor inhibition; improved stability 4: in vitro + in vivo 5: P-gp-mediated DOX efflux 6: indirect – inferred from retention (no direct functional assay in report)	[84]
breast cancer	polymeric micelles – PLA-PEG	1: paclitaxel (PTX) + lapatinib (LAP) 2: LAP inhibits ABC transporters (P-gp, MRPs, BCRP); EGFR blockade; enhanced PTX accumulation 3: ↑ PTX retention in MDR MCF-7; significant tumor inhibition; sustained release 264 h 4: in vitro + in vivo 5: ABC transporters + EGFR 6: indirect – inferred from retention (LAP is known as ABC inhibitor)	[85]
breast cancer	polymer–inorganic hybrid – histidine-modified dextran (DHTD) + Zn-porphyrin (Zn-TPP)	1: doxorubicin (DOX) + paclitaxel (PTX) 2: ROS-triggered DOX release via thioketal; PTX release via Zn-TPP disruption; PDT generates ^1^O_2_; MDR reversal via ROS 3: ↑ drug accumulation in MCF-7/ADR; significant tumor inhibition; synergistic chemo-PDT 4: in vitro + in vivo 5: P-gp + ROS/PDT 6: direct – ROS-mediated P-gp suppression confirmed	[86]
breast cancer	polymeric dendrimer-micelles – mAb 2C5-modified (2C5-MDM)	1: doxorubicin (DOX) + siRNA (MDR1) 2: 2C5 targets cell-surface nucleosomes; siRNA ↓ P-gp 3: 29% P-gp reduction in MDR tumors; ↑ DOX retention; similar tumor reduction to free DOX with less toxicity 4: in vitro + in vivo 5: ABCB1 silencing 6: direct – P-gp reduction quantified	[87]
breast cancer	polymeric micelles – HA-g-cystamine-PLLZ (HA-ss-PLLZ)	1: paclitaxel (PTX) + apatinib (APA) 2: CD44 endocytosis; GSH-redox release; APA inhibits P-gp; chemo synergy 3: ↑ intracellular PTX in MCF-7/ADR; significant tumor inhibition; improved chemosensitivity 4: in vitro + in vivo 5: P-gp-mediated PTX efflux 6: direct – P-gp function + retention	[88]
breast cancer	polymeric (peptide) micelles – transformable chimeric peptide (CTGP)	1: doxorubicin (DOX) 2: cathepsin B cleavage → nanofibers that adhere to cell membrane and restrict DOX efflux 3: ↑ DOX accumulation in MCF-7R (45-fold); 49× anti-MDR vs free DOX; efficient tumor inhibition; prolonged retention 4: in vitro + in vivo (MCF-7R) 5: mechanical barrier to P-gp efflux 6: direct – DOX retention quantified + membrane localization imaging	[89]
breast cancer	polymeric micelles – indomethacin-grafted dextran (IDAC)	1: doxorubicin (DOX) + indomethacin (IND) 2: pH-triggered release; IND ↓ MRP1; Bcl-2/Bax regulation 3: 92.5% TGI in xenograft; ↑ DOX accumulation; effective MRP1 suppression 4: in vitro + in vivo 5: MRP1 suppression 6: direct – MRP1 expression quantified	[90]
colorectal cancer	polymeric mixed micelles – TPGS-based	1: ferulic acid (FA) 2: ↑ solubility/bioavailability; regulates miR-221/TP53INP1 for autophagy/apoptosis; TPGS enhances anticancer activity 3: ↑ FA release vs free; ↓ miR-221, ↑ TP53INP1/Bax/CASP-3; ↑ G2/M arrest in Caco-2 4: in vitro (Caco-2) 5: miR-221 pathway + P-gp 6: indirect – pathway-level regulation; no direct P-gp functional assay	[91]
colorectal cancer	polymeric mixed nanomicelles – VES-CSO/TPGS-RGD	1: bufalin (BU) 2: P-gp efflux inhibition; apoptosis in MDR cells; controlled release 3: ↑ intracellular uptake/cytotoxicity in LoVo/ADR; ↓ P-gp; improved efficacy with lower toxicity in vivo 4: in vitro + in vivo (LoVo/ADR) 5: P-gp expression 6: direct – P-gp expression reported	[92]
chemotherapy-resistant cancer	polymeric oral micelles – quercetin-chitosan (QT-CS) [polymer matrix; chitosan = natural biopolymer]	1: doxorubicin (DOX) 2: ↑ DOX solubility; TJ opening; P-gp efflux evasion 3: 136.9 nm, +16.2 mV; sustained GI release; 2.2× cellular uptake vs free DOX; 10.17× permeability in Caco-2 4: in vitro (Caco-2) 5: P-gp efflux + TJ opening 6: direct – permeability + uptake	[93]
colorectal cancer	polymeric surfactant – Polysorbate 20 (PS20) [non-ionic surfactant, not a NP per se; flag for reclassification]	1: etoposide 2: P-gp inhibition → reduced efflux; ↑ oral absorption 3: ↓ P-gp efflux in Caco-2 and MDCKII MDR1; ↑ oral absorption of etoposide in WT rats (5%/25% PS20); in mdr1a-KO rats no change at 5%, ↓ at 25% 4: in vitro + in vivo (Caco-2; MDCKII-MDR1; rats) 5: P-gp efflux (WT vs KO) 6: direct – efflux ratio + KO control (strongest evidence)	[94]
colorectal cancer	polymer–inorganic hybrids – MSN with polydopamine (PDA) + AS1411 aptamer	1: anti-miR-155 2: targets miR-155; breaks miR-155/NF-κB loop; sensitizes to 5-FU 3: ↓ miR-155 in SW480; ↑ therapeutic efficacy in vitro and in vivo; ↑ sensitivity to 5-FU 4: in vitro + in vivo 5: miR-155/NF-κB axis 6: indirect – operates upstream of P-gp regulation	[33]
colorectal (CRC) + leukemia (HL-60/DOX)	polymer–inorganic hybrids – ZSM-5/KIT-6 and ZSM-5/SBA-15 with polymeric coating (mesoporous silica)	1: verapamil + doxorubicin (DOX) 2: P-gp modulation by verapamil + prolonged release 3: prolonged verapamil release; ↑ intracellular DOX in MDR+; ↑ antitumor activity vs free DOX 4: in vitro 5: P-gp modulation 6: direct – verapamil is a canonical P-gp inhibitor	[95]
colorectal cancer	polymeric NPs – MPEG_2_k-PCL_10_k	1: cabazitaxel (CTX) 2: prevents MDR1-mediated expulsion; improved biocompatibility and bioavailability 3: greater stability, improved antitumor efficacy, lower toxicity than Jevtana 4: in vitro + in vivo 5: MDR1 bypass 6: indirect – inferred from efficacy vs reference formulation	[96]
colon cancer	polymeric NPs – PNPs coated with folate and chitosan (C-FA-PNPs)	1: irinotecan (IRI) + quercetin (QT) 2: FR-targeted delivery; P-gp inhibition by quercetin 3: FR-targeted delivery and P-gp inhibition in colon cancer cells 4: in vitro 5: P-gp-mediated efflux 6: indirect – quercetin known P-gp inhibitor; functional assay not shown	[97]
prostate + breast cancer	polymeric NPs – MePEG-b-PCL (multiple block ratios)	1: docetaxel + paclitaxel 2: P-gp inhibition + ultrasound-assisted cellular uptake 3: restored sensitivity to PTX/DTX in MDCK-MDR 4: in vitro (MDCK-MDR) 5: P-gp-mediated efflux 6: indirect – inferred from restored sensitivity	[98]
breast cancer	polymeric hypoxia-sensitive micelles – PAPD (PEG-azobenzene-PEI-DOPE)	1: doxorubicin (DOX) + siRNA 2: hypoxia-induced PEG removal; PEI charge exposure; siP-gp reduces P-gp 3: ↑ uptake 60%; ↓ P-gp 60%; ≈73% cytotoxicity in MCF7-ADR spheroids; sustained DOX release 4: in vitro (MCF7-ADR spheroids) 5: ABCB1 silencing under hypoxia 6: direct – siRNA knockdown + quantified P-gp reduction	[42]

^a^Abbreviations: HA: hyaluronic acid; DOX: doxorubicin; PTX: paclitaxel; DTX: docetaxel; CTX: cabazitaxel; CUR: curcumin; TPGS: ᴅ-α-tocopheryl polyethylene glycol 1000 succinate; PLGA: poly(lactic-co-glycolic acid); PEG: polyethylene glycol; PCL: poly(ε-caprolactone); PLA: polylactic acid; MSN: mesoporous silica nanoparticle; PDA: polydopamine; GSH: glutathione; ROS: reactive oxygen species; MRP1: multidrug resistance-associated protein 1; BCRP: breast cancer resistance protein (ABCG2); P-gp: P-glycoprotein (ABCB1/MDR1); PDT: photodynamic therapy; PTT: photothermal therapy; TGI: tumor growth inhibition; RI: resistance index; TME: tumor microenvironment; EPR: enhanced permeability and retention; Rh123: Rhodamine 123 efflux assay; WB: Western blot; qPCR: quantitative PCR. P-gp Evidence Type: direct = functional assay (Rh123/calcein-AM/ATPase/silencing) or validated chemical inhibitor; indirect = only inferred from cytotoxicity/retention without orthogonal functional assay.

**3.2.2.1 Structures of polymeric nanoparticles.** Structurally, polymeric nanoparticles can be classified into nanospheres and nanocapsules, each presenting distinctive architectures and drug-release behaviors suitable for prolonged, sustained, or targeted drug delivery [99]. Nanocapsules are vesicular devices that encapsulate an active chemical within a reservoir, enclosed by a polymeric membrane. This form facilitates the encapsulation of both hydrophilic and lipophilic molecules, contingent upon the characteristics of the core and shell materials. Drug release from nanocapsules may transpire through diffusion over the polymer shell, membrane expansion, polymer degradation or erosion, or a combination of these methods [100]. This architecture facilitates regulated and prolonged release, enhanced absorption, and diminished adverse effects. Nanocapsules have been utilized in diverse domains, including medicine, cosmetics, and agriculture.

In contrast, polymeric nanospheres are solid matrix systems in which the active substance is uniformly dispersed throughout the polymer. Unlike nanocapsules, nanospheres do not possess a central cavity, resulting in a more compact and homogeneous structure [101]. Drug release from nanospheres typically occurs via diffusion through the matrix, polymer erosion, or both. This configuration is particularly well suited for extended drug release, enhancing treatment adherence, and has applications in diagnostics, medicine, and agriculture [102]. The choice between nanocapsules and nanospheres is contingent upon various criteria, including the physicochemical characteristics of the medication, the intended delivery route, and the specified release profile. Both techniques have substantial benefits in improving therapeutic effectiveness and reducing side effects, with their optimization being an active field of investigation in medical nanotechnology.

Recent studies have reinforced the relevance of polymeric nanospheres and nanocapsules in the context of MDR therapy, particularly due to their tunability in terms of biodegradability, release kinetics, and capacity for surface functionalization. For instance, Abasian et al. [103] highlighted the role of polymer-based nanocarriers in overcoming MDR through passive and active targeting mechanisms, while Abbasi et al. [104] reported advances in the design of polymeric delivery systems enabling enhanced drug loading and controlled release. Similarly, Cao et al. [105] conducted a comparative analysis of nanospheres versus nanocapsules, demonstrating that nanospheres offer superior sustained-release capabilities, whereas nanocapsules provide higher protection for labile drugs. These recent investigations provide contemporary support for the structural classification presented herein and substantiate the selection of polymeric nanoparticles as versatile and clinically relevant platforms in MDR modulation.

An obvious example are polymeric nanocapsules composed of γ-PGA-*g*-PLGA coated with cholesterol–PEG (C-PEG), which establish a versatile platform to address several mechanisms of MDR. The amphiphilic copolymer has a hydrophobic core that can co-encapsulate doxorubicin, a chemotherapeutic agent, and indocyanine green, a photothermal agent. The C-PEG coating fulfills two essential functions: It extends circulation duration and improves passive tumor accumulation through the EPR effect. It breaks lipid rafts in the cell membrane, reducing P-gp activity and promoting nanoparticle entrance via γ-glutamyltransferase receptor-mediated endocytosis [90].

In MCF-7/MDR cell cultures, this nanoplatform enables significantly higher DOX internalization and nuclear localization compared to the free drug, even prior to external activation. Upon activation with an 808 nm near-infrared (NIR) laser, a localized photothermal effect raises the tumor temperature by over 15 °C, triggering rapid DOX release and inducing cancer cell death without harming healthy tissues. In murine models, the CP2k-DI-NPs nanocomplex effectively suppressed MDR tumor development and caused apoptosis, while exhibiting low systemic toxicity and no noticeable weight loss in the treated mice. Moreover, the chemo-photothermal synergism diminished resistance development and maintained therapeutic efficacy at decreased doses, highlighting the potential of these nanocapsules as an integrated approach for rejuvenating traditional cytotoxic medicines in the management of resistant malignancies [72].

**3.2.2.2 Stimuli-responsive and multifunctional nanocarriers.** While nanospheres and nanocapsules provide structural stability and sustained release, advances in polymer chemistry have enabled the design of stimuli-responsive and multifunctional polymeric nanocarriers capable of engaging tumor-specific triggers and actively reversing P-gp-mediated drug resistance. Polymeric nanoparticles can be functionally designed to respond to tumor-associated stimuli (e.g., acidic pH, elevated glutathione (GSH) levels, presence of reactive oxygen species, or hypoxia) or integrate multifunctional therapeutic capabilities such as P-gp inhibition, redox modulation, photothermal activation, or gene silencing. These systems enable spatiotemporal drug release, enhanced intracellular retention, and the strategic reversal of MDR.

**3.2.2.2.1 Polymeric micelles (pH/redox/ROS-sensitive).** Stimuli-responsive polymeric micelles are self-assembled nanoparticles formed from amphiphilic copolymers, designed to enhance the delivery of anticancer drugs, particularly hydrophobic ones [106]. Their architecture, consisting of a hydrophobic core and a hydrophilic corona, protects the encapsulated drug during systemic circulation and improves solubility. To prevent premature drug release and increase efficacy at the tumor site, these micelles are engineered to respond to internal stimuli such as acidic pH, elevated glutathione levels, specific enzymes, or hypoxic conditions, as well as to external stimuli [106]. Moreover, advanced micelle systems have been developed with responsiveness to multiple stimuli, enabling more controlled and precise drug release. Despite their strong therapeutic potential, challenges remain in terms of long-term stability, biocompatibility, and regulatory approval for clinical applications [107].

Recent investigations into polymeric micelles and multiple stimuli-responsive polymeric nanoparticles have underscored their pivotal role in overcoming MDR in cancer. For instance, Ghezzi et al. [108] offered an in-depth analysis of how polymeric micellar systems interact with the biological milieu, highlighting key parameters such as core stability, corona architecture, and release triggers that directly impact MDR reversal. López Ruiz et al. [109] comprehensively reviewed single and multiple stimuli-responsive polymer particles and noted that dual-trigger systems (e.g., pH + redox) significantly enhance intracellular drug retention and circumvent efflux pumps compared to single stimulus-responsive carriers. More recently, Hou et al. [110] described polymeric nanomedicines engineered for hypoxic tumors that integrate multistimuli responses to deliver therapeutic payloads precisely in tumor microenvironments, thereby addressing both physiological barriers and drug-resistance pathways. These recent data support the comparative framework presented in this review and reinforce the importance of polymeric carrier design that goes beyond structural classification to functional modulation of MDR phenotypes.

Stimuli-responsive polymeric micelles combine a hydrophobic core, which protects the drug, with stimuli-sensitive hydrophilic coronas, enabling drug release and P-gp inhibition precisely at the tumor site. An illustrative instance is the TPGS-IDM system, in which vitamin E-PEG-succinate (TPGS) serves as a competitive inhibitor of P-gp. The ester bond with indomethacin is hydrolyzed under acidic, esterase-abundant endosomal conditions, resulting in the release of paclitaxel. Simultaneously, TPGS diminishes intracellular ATP levels, while indomethacin inhibits MRP1, resulting in elevated ROS levels, mitochondrial impairment, and a marked decrease in the viability of MCF-7/ADR cells, exceeding the impact of free paclitaxel [73].

A different approach utilizes hybrid micelles formed from Pluronic F127 and phenylboronic acid-modified P123, which exhibit sensitivity to increased hydrogen peroxide (H_2_O_2_) concentrations in tumors. The phenylboronate moiety in this system interacts with ROS and diminishes GSH levels, hence promoting the release of DOX. Concurrently, P123 fragments impede P-glycoprotein function. The outcome is an increased intranuclear accumulation of DOX and an up to 88% reduction of tumor development in MCF-7/ADR models, accompanied by minimal systemic damage. These examples illustrate the key to success of integrating three synergistic functions within a single nanocarrier: (i) protection of the cytotoxic agent until release triggered by pH or ROS stimuli, (ii) incorporation of a sensitizer (e.g., TPGS, indomethacin, Pluronic fragments) to deplete energy or inhibit efflux pumps, and (iii) utilization of polymers with an ultra-low critical micelle concentration (CMC) for high circulatory stability [66].

The combination of targeted drug release and energetic sabotage transforms these micelles into modular platforms capable of restoring the efficacy of conventional anticancer drugs against multidrug-resistant tumors, while reducing required doses and systemic toxicity. The immediate challenge is to translate this biochemical synergy into clinical settings, validating improved survival outcomes without additional adverse effects.

**3.2.2.2.2 Multistimuli (redox/pH)-responsive nanoparticles.** Multistimuli-responsive polymeric nanocarriers represent a frontier in precision oncology, integrating both environmental triggers (e.g., pH, redox, and hypoxia) and active functional responses (e.g., efflux inhibition and size/charge modulation). Yoo et al. demonstrated that polymeric micellar systems capable of pH and redox sensitivity achieved markedly improved intracellular retention and efflux-pump bypass in resistant tumor models [111]. Additionally, Hou et al. explored polymer nanomedicines tailored for hypoxic tumors, outlining how these smart carriers can deliver payloads selectively in microenvironments associated with drug resistance [110]. These studies substantiate the rationale for multitrigger polymeric systems and strengthen the comparative framework provided in our classification.

Mechanistically, pH- and redox-responsive polymeric nanocarriers contribute to overcoming P-gp-mediated drug resistance through coordinated intracellular processes. These systems are predominantly internalized via endocytosis, thereby bypassing direct interaction with P-gp efflux pumps located at the plasma membrane [32]. Once internalized, acidic endosomal conditions and elevated intracellular GSH levels trigger nanoparticle destabilization, resulting in rapid intracellular drug release in compartments where P-gp activity is limited [30]. This localized release can transiently increase intracellular drug concentration beyond the efflux capacity of P-gp, enhancing cytotoxicity in resistant cells. Additionally, redox-responsive cleavage of disulfide or diselenide bonds promote endosomal escape and cytoplasmic drug delivery, further reducing drug exposure to efflux mechanisms. In some cases, these processes have also been associated with indirect modulation of P-gp expression or ATPase activity, depending on nanoparticle composition [36].

These devices demonstrate dual responsiveness, integrating both pH and redox sensitivity, which improves targeted selectivity and optimizes antitumor activity. Utilized materials comprise polymers including imine, hydrazone, or ester links that are susceptible to cleavage at low pH, in addition to GSH-sensitive disulfide-containing compounds. Furthermore, the physicochemical characteristics, especially particle size, are meticulously adjusted to enhance performance. Nanoparticles measuring 20–200 nm can reduce rapid renal clearance and are often favorable for tumor accumulation via the EPR effect. However, size alone does not guarantee immune evasion, as nanoparticles within this range may still undergo opsonization and clearance by the mononuclear phagocyte system. Therefore, surface properties such as charge, hydrophilicity, and functionalization play a critical role in determining circulation time and immune recognition. pH-induced aggregation within the tumor microenvironment has been proposed to enhance local retention and drug accumulation. However, this effect is context-dependent as excessive aggregation may reduce tissue penetration and potentially increase clearance. Therefore, achieving a balance between retention and diffusion is essential to optimize therapeutic efficacy [112].

However, the tumor environment is not always homogeneous; not all cancer cells simultaneously present an acidic pH and higher levels of GSH, which are necessary to activate the release of redox/pH-responsive nanoparticles. Therefore, although redox/pH-responsive nanoparticles improve selectivity over single-stimulus systems, this variability leads to incomplete release and insufficient dosing [113]. Therefore, it has been proposed to consider other conditions, such as specific enzymes or hypoxia, to achieve more uniform release [114].

While tumor heterogeneity presents a significant challenge, redox/pH-responsive nanoparticles are a promising approach to controlled drug delivery for cancer treatment. These systems combine high specificity, reduced toxicity, and the potential for individualized treatments. Persistent challenges, particularly tumor heterogeneity, scalability, and clinical application, are driving continuous advances in material design and functionalization, broadening the prospects for next-generation targeted therapies. Examples of redox/pH-responsive nanoparticles are presented in Table 4.

**Table 4:** Multistimuli (pH/Redox/ROS/NIR)-responsive nanoparticle strategies against multidrug resistance.^a^

Type of cancer	Nanoparticles used	1: Encapsulated drug, 2: Mechanism of action, 3: Experimental results, 4: Model, 5: MDR criterion, 6: Stimuli type	Ref.

breast cancer	pH-sensitive PBAE-g-β-CD nanoparticles	1: doxorubicin (DOX) + adjudin (ADD) 2: pH-responsive dual release; ADD ↓ mitochondrial ATP and ↓ P-gp; synergistic apoptosis 3: ↑ intracellular DOX in MCF-7/ADR; ↓ P-gp/XIAP; 73% tumor inhibition in xenografts 4: in vitro + in vivo (MCF-7/ADR) 5: P-gp + XIAP 6: pH	[115]
breast cancer	EphA10 antibody-conjugated pH-sensitive lipoplexes (DOX + siRNA/ePL)	1: doxorubicin (DOX) + MDR1-siRNA 2: pH-sensitive release; EphA10-mediated targeting; siRNA ↓ P-gp; endosomal escape 3: ↑ intracellular DOX in MCF-7/ADR; significant tumor growth inhibition; ABCB1 suppression 4: in vitro + in vivo 5: ABCB1 silencing 6: pH	[116]
breast cancer	pH-sensitive poly(β-amino ester) (PHP) micellar nanoparticles	1: doxorubicin (DOX) 2: acidic pH release; proton-sponge lysosomal escape; P-gp inhibition; ↓ mitochondrial ATP 3: ↑ DOX in MCF-7/ADR; tumor growth inhibition; ↓ P-gp 4: in vitro + in vivo 5: P-gp + ATP 6: pH	[117]
lung cancer	pH/redox cascade-sensitive multiscale nanoparticles (DMA-NPs)	1: podophyllotoxin (PPT; non–P-gp substrate) 2: charge reversal + size shrinkage (170→10 nm); GSH-triggered disulfide release; P-gp bypass via non-substrate payload 3: in TME: ↓ size, ↑ penetration; controlled intracellular release; MDR overcome 4: in vitro + in vivo (A549/PTX) 5: non–P-gp substrate + GSH 6: pH + redox	[118]
colorectal cancer	pH/ROS-sensitive nanoparticles (PLP-NPs)	1: paclitaxel (PTX) + β-lapachone (Lapa) 2: polyhistidine endo-/lysosomal escape; Lapa generates ROS and ↓ ATP → P-gp inhibition; PTX cytotoxicity 3: EPR effect tumor accumulation; ↓ P-gp/ATP; ↑ intracellular PTX; ↑ antitumor response in vitro/in vivo 4: in vitro + in vivo 5: P-gp + ATP 6: pH + ROS	[119]
breast cancer	ROS-responsive nanohybrid – diselenide-crosslinked PAMAM-poloxamer 188 + graphene oxide (ICG/GPP)	1: indocyanine green (ICG) 2: ROS-sensitive ICG release; NIR-triggered PTT + PDT; P-gp suppression 3: ↑ nuclear ICG accumulation in MCF-7/ADR; ↑ cytotoxicity with PDT+PTT under NIR; ↓ P-gp → MDR reversal 4: in vitro (MCF-7/ADR) 5: P-gp + ROS/NIR 6: ROS + NIR	[120]
breast cancer + glioma	protein hybrid nanoplatform (ODP-TH) – transferrin + hemoglobin	1: protoporphyrin IX (PpIX) + doxorubicin (DOX) 2: TFR homotypic targeting; Hb oxygen transport alleviates hypoxia; GSH disulfide release; ↓ MDR1/HIF-1α 3: ↑ DOX accumulation; tumor growth inhibition in breast/glioma; enhanced PDT efficiency; stronger effect vs chemo/PDT alone 4: in vitro + in vivo 5: MDR1 + HIF-1α + PDT 6: redox + NIR (PDT)	[121]
breast cancer	CD44- and mitochondria-targeted HT@ER/PTX nanoparticles	1: paclitaxel (PTX) + encequidar (ER; P-gp inhibitor) 2: CD44 and mitochondrial dual targeting; ER inhibits P-gp; oxidative stress; caspase-3 apoptosis 3: 72.64% tumor inhibition (vs Taxol^®^ 32.36%); prolonged survival; effective P-gp suppression; reduced systemic toxicity 4: in vitro + in vivo 5: P-gp (ER inhibitor) + mitochondrial 6: redox (mitochondrial)	[122]
breast cancer	Y1 receptor ligand-functionalized nanoparticles	1: doxorubicin (DOX) 2: Y1 receptor-mediated targeting; endocytosis-based DOX accumulation; P-gp inhibition 3: ↑ DOX uptake in Y1+ MDR cells; ↑ cytotoxicity/apoptosis vs non-targeted NPs; ↓ systemic toxicity 4: in vitro + in vivo 5: Y1 targeting + P-gp 6: receptor-mediated + pH	[123]
breast cancer	Ir-ss-Qu NPs – self-assembled irinotecan/quinine disulfide-linked	1: irinotecan (Ir) + quinine (Qu) 2: amphiphilic self-assembly; GSH-triggered disulfide break releases Ir + Qu; Qu reduces P-gp 3: IC_50_ 9.1 µM vs 168.7 µM free Ir; ↓ P-gp; 52.48% apoptosis; in vivo TIR 64.10% 4: in vitro + in vivo 5: P-gp + GSH 6: redox	[106]
breast cancer	carrier-free self-assembled nanocrystals – celastrol + DOX	1: celastrol (CST) + doxorubicin (DOX) 2: stable self-assembly; CST inhibits P-gp; ROS/JNK apoptosis; NF-κB suppression; HSF-1 ↓ P-gp 3: ↑ intracellular DOX in MCF-7/ADR; ↑ cytotoxicity; ↑ tumor inhibition; ↑ apoptosis/autophagy vs free drugs 4: in vitro + in vivo 5: P-gp + ROS/NF-κB 6: ROS + redox	[124]
lung cancer	cocrystal-protein anchoring nanococktail (HA-HNRplex)	1: paclitaxel (PTX) + disulfiram (DSF) + cytochrome c 2: triple payload via HA targeting; DSF suppresses P-gp; ↑ intercellular Cyt C; ↑ cleaved-caspase 3; MDR cell apoptosis 3: targeted triple-payload; P-gp suppression; apoptosis pathway activation 4: in vitro 5: P-gp (DSF) + caspase-3 6: pH + redox	[125]

^a^Abbreviations: DOX: doxorubicin; PTX: paclitaxel; PPT: podophyllotoxin; Lapa: β-lapachone; ICG: indocyanine green; PpIX: protoporphyrin IX; ER: encequidar (P-gp inhibitor); Ir: irinotecan; Qu: quinine; CST: celastrol; DSF: disulfiram; Cyt C: cytochrome c; ADD: adjudin; PBAE-g-β-CD: poly(β-amino ester)-grafted β-cyclodextrin; PHP: poly(β-amino ester) pH-sensitive micelles; DMA-NPs: pH/redox cascade-responsive multiscale nanoparticles; PLP-NPs: pH/ROS-sensitive polyhistidine NPs; ICG/GPP: ROS-responsive diselenide–PAMAM–Poloxamer 188/graphene oxide nanohybrid; ODP-TH: transferrin-hemoglobin protein hybrid nanoplatform; HT@ER/PTX: CD44/mitochondria-targeted NPs; HA-HNRplex: HA-anchored cocrystal/protein nanococktail; P-gp: P-glycoprotein (ABCB1); MDR1: multidrug resistance gene 1; HIF-1α: hypoxia-inducible factor-1α; ROS: reactive oxygen species; GSH: glutathione; PTT: photothermal therapy; PDT: photodynamic therapy; XIAP: X-linked inhibitor of apoptosis protein; TIR: tumor inhibition rate; NIR: near-infrared; MCF-7/ADR: doxorubicin-resistant MCF-7; TFR: transferrin receptor; Hb: hemoglobin; EPR: enhanced permeability and retention.

**3.2.2.2.3 DMA-NP cascade nanoparticles.** Multiscale DMA-NP nanoparticles are designed as a versatile delivery technology adept at overcoming the physicochemical obstacles posed by MDR malignancies [118]. Their architecture comprises two essential components, that is, (i) a dendrimer core consisting of podophyllotoxin (PPT) linked by a disulfide bond (PAMAM-ss-PPT) and (ii) a charge-reversible polymeric shell formulated from PEG-PAH-DMA. In the bloodstream (pH 7.4), these nanoparticles have a negatively charged surface, facilitating extended circulation and reducing premature clearance. Upon encountering the acidic tumor microenvironment (pH ≈6.5), hydrolysis of the DMA groups reveals amine groups, leading to a charge reversal to positive. This induces core expulsion and nanoparticle reduction to roughly 10 nm, facilitating deep infiltration into poorly vascularized and hypocellular tumor areas. Upon entering the cell, the increased intracellular GSH concentrations rupture the disulfide bonds, liberating active podophyllotoxin, a cytotoxic compound that does not function as a P-gp substrate. This circumvents efflux via the multidrug resistance pump, facilitating effective microtubule breakdown and subsequent cell death.

This sequential cascade strategy yields remarkable therapeutic outcomes, namely, (i) tenfold higher tumor uptake compared to free PPT, (ii) uniform intratumoral penetration up to 3000 µm, and (iii) 90% tumor growth inhibition in A549/PTX (taxane-resistant lung cancer) models. All outcomes were achieved with a single dose at the maximum tolerated dose, without inducing weight loss or hemolysis. This intelligent nanoparticle design demonstrates that the combination of size shrinkage, charge reversal, and redox-triggered release within a single vector enables efficient nuclear delivery of non-P-gp-substrate drugs, thereby bypassing efflux-mediated resistance without requiring toxic P-gp inhibitors.

It should also be noted that the TME imposes several physical barriers that significantly limit nanocarrier penetration, including a dense stromal matrix, extensive fibrosis, and elevated interstitial fluid pressure, all of which may restrict deep drug diffusion even in the presence of passive EPR-based accumulation. These biomechanical constraints contribute to heterogeneous drug distribution within resistant tumor regions. To overcome such challenges, newer platforms such as DMA-NPs are engineered with cascade activation, wherein pH-triggered charge reversal and subsequent size shrinkage allow for enhanced interstitial penetration and improved transport across the hypovascular and dense stromal regions. This adaptive modulation is particularly relevant in MDR tumors, where limited intratumoral distribution can reduce therapeutic outcomes despite cellular-level efflux inhibition.

**3.2.3 Inorganic nanoparticles: porous silica and metals such as gold, silver, and iron.** Inorganic nanoparticles, including porous silica and metal nanoparticles (gold, silver, and iron), possess unique physicochemical properties that make them highly versatile tools widely employed in a variety of scientific, biomedical, and environmental applications [126]. Porous silica nanoparticles are notable for their high specific surface area and large pore volume, allowing them to encapsulate and transport high loads of active substances, such as drugs, proteins, enzymes, or molecular sensors. These attributes render them particularly appropriate for applications in regulated medication delivery, diagnostic system formulation, and the creation of highly sensitive biosensors [127].

Metallic nanoparticles demonstrate remarkable optical, electrical, and catalytic properties, prompting their incorporation into several domains. Gold nanoparticles are extensively utilized in cancer cell detection, immunoassays, and diagnostic methodologies owing to their biocompatibility, chemical stability, and capacity to traverse biological barriers, such as the blood–brain barrier [128]. Silver nanoparticles, in contrast, are well known for their antimicrobial activity and are being evaluated for use in antimicrobial textiles, self-disinfecting paints, and medical devices such as bactericidal dressings and catheters [129]. Iron nanoparticles, particularly in the form of magnetic oxides, are used for their magnetic properties, which enable easy recovery and reuse [130]. These nanoparticles are applied in environmental remediation, including groundwater decontamination, heavy metal removal, and even in the capture of microplastics. Collectively, these inorganic nanoparticles represent key tools for advancements in nanotechnology, with applications spanning from personalized medicine to environmental protection, underlining their role as fundamental components in the development of future technology. Examples of inorganic nanoparticles are presented in Table 5.

**Table 5:** Inorganic nanoparticles strategies against multidrug resistance.^a^

Type of cancer	Nanoparticle used	Encapsulated drug	Mechanism of action	Experimental results	Ref.

breast cancer	silica-based nanocarrier (formed by electrostatic interaction of APTES and co-condensation with TEOS)	doxorubicin (intercalated with siRNA targeting the MDR-1 gene)	siRNA suppresses P-gp expression, reducing the ability of resistant cells (MCF-7/MDR) to expel doxorubicin. Doxorubicin is pH-sensitive and released accordingly.	This nanocarrier reduces P-gp levels, increases DOX retention, and enhances MCF-7/MDR cell inhibition in vitro by silencing genes and increasing cytotoxicity.	[72]
breast cancer	mesoporous silica nanoparticles	mitomycin C (MMC) and rhodamine 123 (Rho123)	MSNs are internalized by cancer cells, releasing the drug and enhancing efficacy by preventing P-gp-mediated efflux in MCF-7 KCR cells.	MCF-7 and MCF-7 KCR internalized RhoB@MSNs, with higher lysosomal localization in MCF-7 KCR. RhoB@Rho123@MSNs released Rho123 control, while RhoB@MMC@MSNs showed sustained release, reducing cell viability with a lower IC_50_ than free MMC, enhancing efficacy against resistant cells.	[131]
breast cancer	cationic anticancer peptide L-K6-modified mesoporous silica nanoparticles (MSN@L-K6)	doxorubicin (DOX)	- pH-sensitive release of DOX to enhance tumor targeting - L-K6 inhibits P-glycoprotein, leading to increased intracellular accumulation of doxorubicin - electrostatic interactions with neoplastic cells to enhance absorption - sequential drug release: CUR is released initially to inhibit P-glycoprotein, followed by the release of DOX for cytotoxic effects - the pH-sensitive ZIF-8 shell disintegrates in acidic environments - elevated drug loading capacity attributable to mesoporous and microporous architecture	- enhanced DOX accumulation in MCF-7/ADR cells - decreased P-glycoprotein expression, reversing multidrug resistance in vitro and in xenograft models - notable reduction in tumor volume and weight in mice administered MSN@L-K6@DOX	[133]
breast cancer	hybrid mesoporous silica nanoparticles (MSN) with microporous zeolite imidazolate framework-8 (ZIF-8) shell	doxorubicin (DOX) and curcumin (CUR)	- sequential drug release: CUR release first to inhibit P-gp, followed by DOX release for cytotoxicity - pH-sensitive ZIF-8 shell dissolves under acidic conditions - high drug loading capacity due to mesoporous and microporous structure	- enhanced DOX retention and nuclear accumulation in MDR cancer cells (MCF-7/ADR) - significant reduction in P-gp expression - improved cytotoxicity in MDR cells compared to free drugs	[133]
breast cancer	dendrimer-like mesoporous silica nanospheres (MSNs) with hydroxy, amine, thiol, and carboxyl modifications	doxorubicin (DOX)	- large-pore MSNs (9 nm) enhance intracellular DOX accumulation - hydroxylation and carboxylation improve drug penetration - cellular uptake primarily via clathrin-mediated endocytosis - increased intracellular drug retention due to non-P-gp substrate properties	- hydroxy- and carboxyl-modified MSNs significantly increased DOX accumulation in MCF-7/ADR cells - enhanced cytotoxicity compared to free DOX - improved apoptosis induction and inhibition of cell proliferation - significant tumor inhibition in xenograft models	[134]
breast cancer	hyaluronic acid-coated, pH/redox dual-responsive mesoporous silica nanoparticles (HPMSNs)	doxorubicin (DOX) and GCN5 siRNA	- CD44-mediated targeting enhances drug internalization - pH/redox dual-responsive nanocarrier releases DOX and siRNA in response to tumor microenvironment - siGCN5 downregulates P-gp epigenetically, increasing DOX retention	- increased DOX accumulation in CD44-overexpressing MDR cells - significant tumor growth inhibition (77%) in xenograft models - almost complete abolition of P-gp-mediated drug resistance - reduced systemic toxicity of DOX	[135]
breast cancer	ᴅ-α-tocopheryl polyethylene glycol 1000 succinate (TPGS)-functionalized mesoporous silica nanoparticles (MSNs)	doxorubicin (DOX)	- pH-sensitive drug release enhances DOX accumulation in tumor cells - TPGS inhibits P-gp, preventing DOX efflux and reversing MDR - enhanced endocytosis via clathrin-mediated and caveolae-dependent pathways - prolonged circulation and tumor-specific targeting due to EPR effect	- tenfold increase in cytotoxicity compared to free DOX in MCF-7/ADR cells - significant tumor inhibition and improved drug retention in xenograft models - higher intracellular DOX accumulation compared to non-functionalized MSNs - reduced systemic toxicity and improved biocompatibility	[136]
gastric carcinoma	silica nanoparticles modified with hyaluronic acid (HA-SiLN/QD)	quercetin and doxorubicin (DOX)	- modification with hyaluronic acid (HA) for recognition of CD44-overexpressed receptors in GC cells - quercetin reduces the expression of Wnt16 and P-glycoprotein (P-gp), remodeling the tumor microenvironment and reversing MDR. - sustained release of DOX to improve its antitumor activity	- nanoparticles with preferred size and sustained release properties - higher selective uptake and retention of DOX in SGC7901/ADR cells (MDR-resistant) compared to single-drug delivery systems (HA-SiLN/D) - significantly improved antitumor efficacy in mice models with SGC7901/ADR tumors compared to single-drug delivery systems (HA-SiLN/Q and HA-SiLN/D)	[137]
colon cancer	functionalized porous silica nanoparticles	cytochrome c (enzyme), AS1411 aptamer, and anticancer drug	- supra-assembly of silica nanoparticles for high cytochrome c loading, AS1411 aptamer, and drug - pH-responsive intracellular release - triple therapy: suppression of P-gp, catalytic action of cytochrome c, and anticancer activity of the drug and the aptamer	- high loading efficiency and controlled release - 92% cell death in chemotherapy-resistant HCT116 cells in vitro - in vivo toxicity studies showed a favorable safety index for cancer therapy	[138]
colorectal cancer	V-doped hollow mesoporous silica (VSi-BP@HA)	BPTES (glutaminase inhibitor)	inhibition of glucose and glutamine metabolism; reversal of chemotherapy and immunotherapy resistance	33% tumor eradication in murine models; synergy with anti-PD1	[139]
breast cancer	mesoporous silica nanoparticles (MSNs) functionalized with amines and coated with hyaluronic acid conjugated to PEG-PLGA (HA@TQ-DOX-MSN)	thymoquinone (TQ) and doxorubicin (DOX)	- coencapsulation and simultaneous release of TQ and DOX - interference with resistance mediated by MDR-1/miR-298 - reduction of drug efflux through inhibition of ABC transporters (P-gp1/MDR-1)	- higher intracellular drug retention in DOX-resistant MDA-MB-231 cells (231R) - significant reduction in drug efflux - resensitization of 231R cells to DOX through the combination of TQ and DOX	[140]
lung cancer	cancer cell membrane (CCM)-coated silica (SLI) nanoparticles	miR495 with doxorubicin (DOX)	overcoming MDR by inhibiting P-glycoprotein (P-gp)	the MDR of cancer cells was overcome through downregulation of P-gp expression using miR495	[141]
breast cancer	NaY(Mn)F4:Yb/Er UCNP associated with TPGS	doxorubicin (DOX) + TPGS (polyethylene glycol dodecyl sulfate)	caveolin- and clathrin-dependent endocytosis, while TPGS enhances drug accumulation, inhibits P-gp, causes mitochondrial depolarization, decreases ATP, induces oxidative stress, and triggers apoptosis in resistant cells	in vitro: conjugation with TPGS enhances DOX accumulation in cells in vivo: reduction of tumor size to 10% of the control and improved apoptosis in tumor cells, while showing minimal systemic and organ toxicity	[142]
breast cancer	HA-MIL-125@DVMA (hollow MIL-125 metal-organic framework coated with hyaluronic acid)	doxorubicin (DOX) in prodrug form DV and 3-methyladenine (3-MA)	inhibition of P-gp to prevent DOX efflux and suppression of autophagy via 3-MA, enhancing intracellular drug accumulation and reversing tumor resistance.	In vitro, HA-MIL-125@DVMA enhanced DOX uptake, inhibited drug efflux, and reduced viability in MCF-7/ADR cells. In vivo, it suppressed tumor growth without body weight loss or organ toxicity.	[143]
breast cancer	magnetic nanoparticles (MNP) loaded with dihydroartemisinin (MNP-DHA)	dihydroartemisinin (DHA), an artemisinin derivative with peroxide groups	generation of reactive oxygen species via Fenton-like reaction catalyzed by ferrous ions released from MNPs in the acidic tumor microenvironment, inducing apoptosis and overcoming MDR by preventing P-gp-mediated drug efflux	In vitro, MNP-DHA showed higher cytotoxicity in MCF-7/ADR cells with an IC_50_ of 7.76 µg/mL (vs 26.10 µg/mL for free DHA), significantly improving growth inhibition. In vivo, it effectively suppressed tumor growth, demonstrating potential for treating challenging breast cancers.	[144]
breast cancer	silver nanoparticles (Ag NPs) functionalized with a peptide	doxorubicin (DOX)	Silver nanoparticles (Ag NPs) induce ER stress and immunogenic cell death (ICD). Doxorubicin conjugated with CB5005 peptide enhances cellular penetration, nuclear accumulation, and reduces P-gp drug efflux, improving biocompatibility.	Ag-TF@PDOX activates ER stress, enhances chemotherapy, reduces P-gp expression, and overcomes drug resistance in MCF-7/ADR. The dual PAI/MRI system guides precision therapy, inhibiting tumor growth and triggering an immune response.	[145]
breast cancer	polyethyleneimine (PEI) and CuTCPP (copper tetrakis (4-carboxyphenyl) porphyrin)-modified nanoparticles	paclitaxel (PTX)	tLyP-1 peptide enables selective nanoparticle uptake in tumor cells. PEI nanoparticles release PTX, and CuTCPP removes GSH and aids photoacoustic imaging. PD-1/PD-L1 inhibition enhances immune response and reduces P-gp, improving therapy.	enhances PTX accumulation and release in acidic environments, increasing cytotoxicity in PTX-resistant cells reduces tumor growth in animal models, with better results using TPP@PTX-CuTCPP activates immune response and controls primary and metastatic tumor growth	[146]
breast cancer	gold nanoparticles (AuNPs) with average sizes of 4.1 and 5.4 nm	ANS and 6-MP	gold nanoparticles modified with ANS and 6-MP reduce P-gp-mediated drug resistance and enhance intracellular drug accumulation by preventing P-gp recognition	Larger nanoparticles (5.4 nm) reduce drug resistance in MCF-7/ADR cells, enhancing drug accumulation and anticancer activity. The critical size for overcoming MDR is between 4.1 and 5.4 nm, with significant metabolic pathway differences observed.	[147]
breast cancer	gold nanorods (GNRs) conjugated with biotin-polyethylene glycol (Biotin-PEG)	paclitaxel (PTX) and curcumin (CUR)	- dual-drug conjugation onto GNRs via robust Au–S bonds - controlled release triggered by glutathione and esterase in tumor cells - photothermal therapy enhances intracellular drug release - CUR inhibits P-gp expression to enhance PTX retention	- increased intracellular PTX and CUR retention at a precise 1:1 ratio - significant MDR reversal and enhanced cytotoxicity in MCF-7/ADR cells - stronger apoptotic effect compared to free PTX/CUR mixture - reduction in P-gp expression, leading to improved drug accumulation	[148]
colorectal cancer	gold nanorod core with triple-layer coating (GNRs/mSiO_2_/PHIS/TPGS)	doxorubicin	- endo-/lysosomal escape via PHIS - inhibition of P-gp with TPGS to increase intracellular retention - pH- and NIR-controlled release	greater intracellular accumulation of DOX and increased cytotoxicity in MDR SW620/Ad300 cells; greater efficacy in murine models without significant systemic toxicity	[149]
colorectal cancer	selenium-doped manganese phosphate nanoparticles (Se-MnP NPs)	oxaliplatin (OX) loaded in Se-MnP NPs	- induction of oxidative stress via activation of the STAT3/JNK pathway - Fenton-like reaction mediated by Mn^2+^ - inhibition of ABC transporters (ABCB1, ABCC1, ABCG2) - induction of apoptosis via caspases	greater accumulation of OX in resistant cells significant reduction in tumor growth in mice reversal of MDR	[150]
prostate cancer	magnetic nanoparticles with polyethylene (PEI) and polyethylene glycol (PEG) layers	miRNA-205	reintroduction of miR-205 to reverse docetaxel resistance	cells treated with miRNA-205-NPs + docetaxel had greater apoptosis, confirming the synergistic effect	[151]
multiple types of cancer that are resistant to multiple drugs	selenium nanoparticles (SeNPs) tellurium nanoparticles (TeNPs)	doxorubicin (DOX), cisplatin (CIS), paclitaxel (PTX), 5-fluorouracil (5-FU), vincristin (VCR), irinotecan	inhibition of P-gp efflux pumps and increased apoptosis	inhibition of metastasis and reduction of drug resistance	[152]
various types of cancer	metal nanoparticles boron and nitrogen nanocompounds with anti-cancer activity catalytic nanoparticles biodegradable nanoparticles are capable of capturing oxygen	therapeutic nanomaterials	generation of reactive oxygen species and modulation of MDR	anti-cancer effect without the need for additional drugs	[153]
breast cancer	NO-responsive liposomal gold@copper sulfide yolk–shell nanoparticles (ADLAu@CuS YSNPs)	doxorubicin (DOX) and ʟ-arginine (L-Arg)	- NO release to inhibit P-gp expression, reducing MDR - sequential drug release: NO release precedes DOX to optimize the tumor microenvironment - photothermal and photodynamic effects via NIR laser irradiation	- enhanced DOX accumulation and retention in MCF-7/ADR cells - significant reduction in P-gp expression - effective tumor inhibition in xenograft models with low systemic toxicity	[154]
breast cancer	beta-cyclodextrin (β-CD)-functionalized Fe_3_O_4_/hydroxyapatite (HAPA) nanocomposites	doxorubicin (DOX) and curcumin (CUR)	- magnetic field-assisted targeting via Fe_3_O_4_ nanoparticles - P-gp inhibition by CUR to enhance DOX accumulation - reduction of protein corona formation for improved tumor targeting - pH-dependent sustained release of DOX and CUR	- increased DOX accumulation in tumor tissue - enhanced cytotoxicity in MDR breast cancer cells (MCF-7/adr) - reduced P-gp expression, leading to improved drug retention - magnetic field targeting improved tumor accumulation but did not significantly enhance therapeutic efficacy over free DOX	[155]
breast cancer	Fe_3_O_4_@SiO_2_-P123/PTX-ZnPc nanoparticles (FSP-PTX-ZnPc NPs)	paclitaxel (PTX) and zinc(II) phthalocyanine (ZnPc)	- magnetic targeting and enhanced permeation and retention (EPR) effect to improve tumor accumulation - photodynamic therapy (PDT) using ZnPc to generate reactive oxygen species and induce apoptosis - inhibition of P-gp-mediated drug efflux, increasing PTX retention - dual chemotherapy and PDT for MDR reversal	- increased intracellular drug uptake and tumor retention in MCF-7/PDR cells - significant tumor inhibition and enhanced apoptosis in xenograft models - effective penetration of the blood–brain barrier for potential brain cancer therapy - reduced systemic toxicity compared to free PTX	[156]

^a^Abbreviations: APTES, (3-aminopropyl)triethoxysilane; TEOS, tetraethyl orthosilicate; P-gp, P-glycoprotein; MDR, multidrug resistance; DOX, doxorubicin; siRNA, small interfering RNA; MMC, mitomycin C; Rho123, rhodamine 123; CUR, curcumin; MSN, mesoporous silica nanoparticle; ZIF-8, zeolitic imidazolate framework-8; HA, hyaluronic acid; HPMSN, HA-coated pH/redox-responsive MSN; GCN5, general control nonderepressible 5; TPGS, ᴅ-α-tocopheryl polyethylene glycol 1000 succinate; CD44, cluster of differentiation 44; HA-SiLN, HA-modified silica lipid nanoparticle; D, doxorubicin-loaded; Q, quercetin-loaded; Cyto c, cytochrome c; AS1411, nucleolin-targeting aptamer; VSi-BP@HA, vanadium-doped hollow silica coated with HA; BPTES, glutaminase inhibitor; UCNP, upconversion nanoparticle; PDOX, peptide-modified DOX; ER, endoplasmic reticulum; ICD, immunogenic cell death; PEI, polyethyleneimine; CuTCPP, copper tetrakis(4-carboxyphenyl)porphyrin; PTX, paclitaxel; PTT, photothermal therapy; MOF, metal-organic framework; DV, DOX-valine prodrug; 3-MA, 3-methyladenine; MNP, magnetic nanoparticle; DHA, dihydroartemisinin; Ag NPs, silver nanoparticles; ANS, 2-(9-anthracenylmethylene)hydrazine-carbothioacetamide; 6-MP, 6-mercaptopurine; GNRs, gold nanorods; PHIS, poly(histidine); Se-MnP NPs, selenium-doped manganese phosphate nanoparticles; OX, oxaliplatin; SF NPs, super-organic framework nanoparticles; PBDF NPs, PLGA-SH/gold nanorod hybrid nanoparticles; IONP-Fol, folate-functionalized iron oxide nanoparticles; miRNA, microRNA; TeNPs, tellurium nanoparticles; CIS, cisplatin; 5-FU, 5-fluorouracil; VCR, vincristine; ADLAu@CuS YSNPs, NO-responsive liposomal gold@copper sulfide yolk–shell nanoparticles; β-CD, beta-cyclodextrin; HAPA, hydroxyapatite; ZnPc, zinc(II) phthalocyanine.

**3.2.3.1 Mesoporous silica nanoparticles.** Hyaluronic acid-coated mesoporous silica nanoparticles (HPMSNs) exemplify how the co-delivery of chemotherapy and gene-silencing agents can effectively reverse P-gp-mediated MDR. These nanocarriers feature a mesoporous silica core loaded with DOX and sealed by a PEI-PEI-β-cyclodextrin “gatekeeper”, which also serves to anchor siRNA targeting GCN5, an acetyltransferase responsible for the epigenetic activation of the MDR1 gene. Following CD44 receptor-mediated endocytosis, the acidic and reductive endosomal environment triggers the dual pH/redox-responsive mechanism, opening the pores and enabling the simultaneous release of DOX and siGCN5. Silencing GCN5 substantially diminishes histone acetylation at the MDR1 promoter, resulting in a notable downregulation of P-gp expression. The result is a significantly improved intracellular retention of DOX. This method resulted in 77% tumor growth inhibition in MCF-7/ADR xenograft animals, with nearly total eradication of P-gp and no indications of cardiotoxicity or weight reduction. The results validate that functionalized mesoporous silica nanospheres serve as secure and highly efficient platforms for integrating chemotherapy with RNA interference, providing a robust strategy to surmount MDR in cancer treatment [139].

**3.2.3.2 Metallic nanoassemblies.** Metallic nanoassemblies offer a dual-function strategy against MDR: They serve both as drug carriers and as inducers of lethal oxidative stress. In the case of gold nanoparticles (AuNPs), Jiang et al. demonstrated that coating 5.4 nm AuNPs with the thiol-containing drugs ANS and 6-mercaptopurine (6-MP) generates a conjugate too large to be effluxed by P-gp [147]. This size-based exclusion resulted in high intracellular accumulation and a reduction of the resistance index from 5 to approximately 2, without requiring co-administration of external P-gp inhibitors. This highlights how the “critical dimension” of metal nanoparticles can enable them to bypass efflux mechanisms and deliver cytotoxic agents directly to tumor cells [147]. Importantly, this concept of a “critical size” extends beyond gold systems and applies broadly to other inorganic nanomaterials, such as mesoporous silica, silver, and iron oxide nanoparticles [127]. Empirical studies have shown that nanoparticles within the 10–200 nm range achieve an optimal balance between circulation time, tumor accumulation via the EPR effect, and evasion of macrophage uptake [132]. For mesoporous silica nanoparticles, diameters between 50 and 150 nm maximize intratumoral diffusion and cellular uptake [127], whereas particles smaller than 10 nm are rapidly cleared renally, and those larger than 200 nm are sequestered by the reticuloendothelial system [147]. Thus, the “critical size” represents a general biophysical constraint determined by tumor vasculature and nanoparticle surface interactions, which collectively influence biodistribution and resistance evasion.

When the metallic core itself participates actively in tumor biochemistry, the therapeutic benefits are further amplified. For instance, folate-conjugated iron oxide nanoparticles co-loaded with doxorubicin and the PARP inhibitor olaparib combine multiple functionalities, namely, folate receptor targeting, T_2_ MRI contrast enhancement, and ROS generation via Fenton-like reactions from the iron core. This multifunctional approach enhances DNA cleavage by olaparib, potentiated by DOX and oxidative stress from the iron, leading to increased apoptosis and inhibited tumor migration in resistant prostate cancer models, while exhibiting lower cardiotoxicity compared to standard chemotherapy [157]. In another example, a Ag-TF@PDOX nanocomposite integrates silver nanoparticles within a tannic acid–ferric ion (TF) network and a peptide-conjugated DOX (CB5005). The silver core induces endoplasmic reticulum (ER) stress, consuming ATP and downregulating P-gp expression. The ATP-dependent disassembly of the complex leads to the release of DOX, while the CB5005 peptide guides nuclear localization [145]. Simultaneously, excessive ROS production and exposure to damage-associated molecular patterns induce immunogenic cell death, hence diminishing metastasis in multidrug-resistant breast cancer models. Collectively, these findings illustrate that noble and transition metal-based nanoparticles, when suitably functionalized, perform as targeted carriers (“smart taxis”) as well as “redox amplifiers” and efflux pump modulators. This combination presents a promising opportunity for re-sensitizing resistant tumors to standard chemotherapeutic drugs [58].

**3.2.4 Nanoparticles based on natural biopolymers.** Nanoparticle-based therapies have transformed contemporary medicine by enabling the precise and targeted delivery of pharmaceuticals. These nanoparticles, generally measuring between 1 and 100 nm, can be produced from either natural or manufactured materials. Their applications have notably broadened across domains including tissue engineering, medication delivery, wound healing, and medical diagnostics. Their high surface-to-volume ratio, ability to interact with cells, and potential for surface functionalization make them highly effective and versatile tools for advanced therapies [158].

Natural biopolymers, including proteins (e.g., albumin, collagen, gelatin, and silk fibroin) and polysaccharides (e.g., chitosan, alginate, cellulose, starch, and hyaluronic acid) have shown great promise as nanoparticle materials. These substances are biodegradable, biocompatible, non-toxic, and generally do not elicit strong immune responses. Due to their functional groups, they can be chemically modified or conjugated with bioactive molecules to improve stability, solubility, and controlled drug release. However, their inherent low mechanical strength often necessitates crosslinking or incorporation into support structures [159]. Natural biopolymer-based nanoparticles are currently being explored for a wide range of therapeutic applications, including cancer treatment, infection control, tissue regeneration, oral insulin delivery, and smart wound dressings. When combined with three-dimensional scaffolds or hydrogels, they can create multifunctional platforms capable of sustained and localized release of bioactive agents. Nonetheless, further research is needed to fully understand the cell signaling pathways involved and to conduct comprehensive clinical studies assessing the safety and efficacy of these materials. Only then can their integration into regenerative medicine be realized on a broader clinical scale. Examples of nanoparticles based on natural biopolymers are presented in Table 6.

**Table 6:** Nanoparticles based on natural biopolymers against multidrug resistance.^a^

Type of cancer	Nanoparticle used	1: Encapsulated drug, 2: Mechanism of action, 3: Experimental results, 4: Model, 5: MDR criterion, 6: Biopolymer role	Ref.

triple-negative breast cancer (TNBC)	hyaluronic acid (HA)–carboxymethyl chitosan (CMC) nanodroplets (HSC-NDs)	1: doxorubicin (DOX) 2: pH/redox-responsive release; UTMD improves penetration; ↑ ROS-driven apoptosis; ↓ P-gp; ↓ EMT 3: ↑ intracellular DOX in MDR TNBC; significant tumor inhibition; ↓ P-gp and MDR mechanisms; improved imaging 4: in vitro + in vivo (MDA-MB-231/ADR) 5: P-gp + EMT + ROS 6: biopolymer matrix – HA + chitosan core forms the nanodroplet	[160]
breast cancer	celastrol-conjugated chitosan oligosaccharide (Cel-CSO) nanoparticles	1: celastrol (Cel) + paclitaxel (Taxol) 2: pH- and time-dependent release; inhibits P-gp, HIF-1α, TUBB3, Tau; apoptosis via PI3K/AKT/NF-κB/HIF-1α suppression 3: ↑ Taxol accumulation in MDR breast cells; ↓ P-gp/HIF-1α; tumor inhibition in xenografts; reduced systemic toxicity 4: in vitro + in vivo (MCF-7/Taxol) 5: P-gp + HIF-1α 6: biopolymer matrix – chitosan oligosaccharide backbone	[161]
breast cancer	chitosan/β-cyclodextrin (CS/CD) nanoparticles	1: DNAzyme targeting MDR1 mRNA 2: DNAzyme cleaves MDR1 mRNA → ↓ P-gp; chitosan ↑ transfection; pH-sensitive release 3: 22-fold resistance decrease in MCF-7/DR; ↓ MDR1 mRNA (RT-qPCR); ↑ Rh123 accumulation; ↑ cytotoxicity 4: in vitro (MCF-7/DR) 5: MDR1 mRNA (qPCR + Rh123) 6: biopolymer matrix – CS/CD nanoparticle	[162]
MDR cancer	gold nanoparticles stabilized with carboxymethyl chitosan (CMC) and decorated with PEG	1: doxorubicin (DOX) 2: sustained pH-dependent release; overcomes P-gp; PEG ↑ circulation 3: 71.2 nm; DOX loading 73.14%; antiproliferative vs A549; ↓ clearance, ↑ t_1/2_ in rats; ↓ efflux ratio in Caco-2 4: in vitro + in vivo (A549; Caco-2; rats) 5: efflux ratio 6: biopolymer coating on inorganic core – AuNP core + CMC/PEG coating	[163]
breast cancer	GMO (glyceryl monooleate) nanostructures stabilized with poloxamer 407 (F127) or polysorbate 80	1: doxorubicin (DOX) 2: prolonged DOX release; poloxamer 407 inhibits P-gp; ammonium sulfate loading ↑ encapsulation 3: 80% DOX encapsulation; sustained release (70–85%, 72 h); ↑ cytotoxicity vs MCF-7/MDA-MB-231; high uptake 4: in vitro (MCF-7; MDA-MB-231) 5: P-gp (poloxamer mechanism) 6: biopolymer-stabilized lipid NPs – lipidic GMO core + poloxamer 407 (synthetic, not biopolymer; FLAG: borderline)	[76]
colorectal cancer	PEG-curcumin/DOX nanoparticles (PEGCRC/DOX NPs)	1: doxorubicin (DOX) 2: ↑ intracellular DOX in MDR; P-gp efflux inhibition; apoptosis induction 3: ↑ DOX accumulation; efflux protein suppression; significant tumor growth reduction in xenograft 4: in vitro + in vivo 5: P-gp efflux 6: biopolymer-conjugate matrix – PEG-curcumin conjugate (curcumin = natural product, PEG = synthetic)	[164]
lung cancer	galactoxyloglucan nanoparticles (PST)	1: paclitaxel (PTX) 2: natural galactoxyloglucan-based PTX NP to overcome MDR and induce apoptosis in resistant lung cancer cells 3: drug efflux overcome; cell death induced in resistant lung cancer cells 4: in vitro 5: PTX efflux 6: biopolymer matrix – natural galactoxyloglucan (plant polysaccharide)	[165]

^a^Abbreviations: HA: Hyaluronic acid; CMC: Carboxymethyl chitosan; HSC-NDs: Hyaluronic acid–carboxymethyl chitosan nanodroplets; UTMD: Ultrasound-targeted microbubble destruction; ROS: Reactive oxygen species; P-gp: P-glycoprotein; TNBC: triple-negative breast cancer; HA: hyaluronic acid; CMC: carboxymethyl chitosan; HSC-NDs: HA–CMC nanodroplets; DOX: doxorubicin; Cel-CSO: celastrol–chitosan oligosaccharide NPs; PI3K/AKT/NF-κB: signaling pathway; HIF-1α: hypoxia-inducible factor-1α; TUBB3: class III β-tubulin; CS/CD: chitosan/β-cyclodextrin; PEG: polyethylene glycol; AuNPs: gold nanoparticles; GMO: glyceryl monooleate; F127: Pluronic/Poloxamer 407; TPGS: ᴅ-α-tocopheryl polyethylene glycol 1000 succinate; LAP: Laponite; Rh123: Rhodamine 123 efflux assay; MDR1: multidrug resistance gene 1.

**3.2.4.1 Hyaluronic acid or chitosan nanoparticles.** Hyaluronic acid (HA)-based nanoparticle systems are increasingly leveraging dithiol-sensitive bonds to trigger site-specific drug release within MDR cells. A recent example are the HA-SS-CMC nanodroplets developed by Xiao et al., which features a disulfide-linked HA–chitosan backbone encapsulating perfluorohexane (PFH) and DOX. In the acidic, GSH-rich TME, disulfide bonds are cleaved, leading to the release of DOX and the decapsulation of PFH. Subsequent ultrasound irradiation causes PFH vaporization and acoustic cavitation, which disrupts endosomal membranes, reduces intracellular GSH, increases ROS, and ultimately downregulates P-gp expression while inhibiting the epithelial–mesenchymal transition (EMT) phenotype. In MDA-MB-231/ADR models, this approach demonstrated a sevenfold increase in intratumoral accumulation, 82% tumor inhibition, a decrease in the Bax/Bcl-2 ratio, and better ultrasound imaging for therapy guiding [160].

An approach utilizing dual chemotherapy are the HA-SS-PLLZ micelles formulated by Zhang and coworkers. These redox-responsive micelles discharge paclitaxel and the anti-angiogenic drug apatinib following disulfide breakage. Co-delivery facilitates synchronized transport, saturating P-glycoprotein, inhibiting EGFR signaling, and depleting ATP, therefore diminishing the resistance index from approximately 25 to nearly 1 in MCF-7/ADR cells and achieving 73% tumor reduction with minimum systemic damage [88].

A chitosan-based method involves the formation of stable, redox-sensitive micelles from quercetin–chitosan conjugates, which function as drug transporters and permeability enhancers. The quercetin moiety inhibits P-glycoprotein ATPase, disrupts tight junctions, and synergistically enhances the cytotoxicity of concurrently administered doxorubicin or paclitaxel upon release. In vitro investigations demonstrate that these micelles decrease the IC_50_ of DOX four- to fivefold in MCF-7/ADR cells, whereas oral administration enhances bioavailability twofold without indications of hemolysis or liver toxicity. The dynamic bond between the chitosan backbone and quercetin ensures ROS/GSH-triggered release while transforming the carrier itself into an efflux pump inhibitor, making it a promising platform for oral or combination therapies requiring epithelial penetration [93]. Taken together, these three studies reinforce the concept that natural polymers equipped with cleavable linkages, whether CD44-targeted HA or bioadhesive chitosan, provide a dual mechanism of action, that is, precise, targeted delivery and strategic inhibition of P-gp, offering a compelling direction for precision nanotherapies against multidrug resistance.

Based on these examples and other orally administered nanocarriers, it is important to note that oral nanocarriers face multiple gastrointestinal obstacles that can reduce both bioavailability and P-gp reversal, including proteolytic/esterase-driven degradation, a dynamic mucus barrier that filters and clears particulates, variable pH (acidic stomach to near-neutral intestine), tight junctions limiting paracellular transport, and apical P-gp efflux in enterocytes. Formulation strategies to address these challenges include pH-selective protection (enteric or acid-stable shells) to transit the stomach and disassemble in the small intestine, mucus navigation either via mucoadhesive chitosan/derivatives (prolonging residence and transiently loosening tight junctions) or mucus-penetrating hydrophilic coronas (e.g., PEG-like) to reduce adhesive trapping; stimuli-responsive chemistries (redox/pH-cleavable linkers) to trigger release at the epithelial surface or within enterocytes, and localized, transient P-gp modulation through co-delivered functional excipients such as quercetin (competitive inhibitor/antioxidant) or TPGS (ATP depletion/P-gp inhibition). Additional levers include permeation enhancers and lipid-rich architectures that favor lymphatic uptake, thereby mitigating first-pass metabolism. For quercetin–chitosan micelles specifically, the chitosan interface can enhance epithelial contact and paracellular transport, while quercetin provides on-site efflux modulation to increase intracellular drug retention. Rigorous evaluation should pair Caco-2/MDCK transport and efflux ratios with biorelevant pharmacokinetics to confirm that oral exposure gains translate into durable MDR reversal without off-target P-gp suppression.

**3.2.4.2 Advanced hybrid nanoparticles with cell membranes.** In recent decades, cell membrane-coated nanoparticles (CNPs) have emerged as a groundbreaking biomedical platform, enabling the creation of safe, targeted, and multifunctional therapies. These nanoparticles are fabricated by cloaking synthetic cores, typically composed of polymers, lipids, or inorganic materials, with natural cell membranes, endowing them with the ability to mimic complex cellular functions such as immune evasion, homotypic targeting, and neutralization of biological threats. This biomimetic approach improves the therapeutic efficacy of nanomedicine by overcoming various constraints inherent in traditional nanoparticle systems, such as fast clearance, nonspecific accumulation, and immune recognition [166].

Recent advancements have resulted in the creation of hybrid CNPs, wherein membranes from several cell types are amalgamated to integrate complimentary biological functions into a singular nanoparticle. For example, hybrid nanoparticles coated with red blood cell (RBC) membranes, which confer long circulation and immune camouflage, fused with platelet membranes, known for their strong adhesion and targeting capabilities, result in nanocarriers that achieve both prolonged systemic retention and enhanced target specificity. This approach holds great promise for the treatment of cardiovascular diseases, cancer, and inflammatory conditions [167].

Other innovative hybrids include combinations of cancer cell membranes with RBC or macrophage membranes, enabling the nanoparticles to simultaneously target tumors via homotypic recognition and evade immune detection. Such systems not only enhance drug delivery to pathological sites but also provide opportunities for personalized therapy, immune modulation, and real-time diagnostics [168].

**3.2.4.2.1 Cancer cell membrane-coated nanoparticles.** Coating silica nanoparticles with tumor cell membranes offers an all-in-one strategy to bypass extracellular barriers and overcome P-gp-mediated drug resistance. In the study by He et al., aminated silica cores co-loaded with DOX and miR-495 were coated with membranes derived from lung cancer cells, generating a homologous nanocarrier designated as CCM/SLI/miR-DOX [141]. The cancer cell membrane provides tumor-specific antigens that facilitate homotypic targeting and protect the nanocarrier from hepatosplenic clearance, enhancing its accumulation at the tumor site. Once internalized, miR-495 silences GCN5, a histone acetyltransferase that regulates MDR1 gene transcription, thereby reducing P-gp expression. The silica core concurrently facilitates the persistent release of DOX within the TME. The treatment outcomes are remarkable, namely, (i) a 1.6-fold increase in intracellular DOX uptake, (ii) more than 50% decrease in P-gp expression relative to controls, (iii) gradual volume diminution in three-dimensional multicellular tumor spheroids, and (iv) nearly total inhibition of xenograft proliferation in mouse models, accompanied by negligible body weight loss. These results significantly exceeded those obtained from DOX or miR-495 monotherapies, validating the synergistic advantage of co-delivery and tumor membrane cloaking.

**3.2.4.2.1.1 Biomimetic nanoparticle manufacturing and regulatory challenges.** Despite their remarkable potential for tumor-specific targeting and immune evasion, cell-membrane-coated nanoparticles (CNPs) still face significant translational barriers. The sourcing of biological membranes, derived from RBCs, platelets, macrophages, or tumor cells, introduces variability in protein composition, sterility, and immunogenicity, complicating large-scale production and clinical standardization. Recent studies highlight the necessity of consistent proteomic and lipidomic profiling to ensure reproducible antigen expression and biological functionality across batches [166]. From a regulatory perspective, these biomimetic nanosystems occupy a gray zone between biologics and nanomedicines, currently lacking harmonized Good Manufacturing Practice frameworks. Therefore, scalable purification, standardized characterization, and validated sterility protocols are indispensable prerequisites for clinical translation. Cancer cell-derived membranes, when combined with therapeutic miRNAs, can turn the very mechanisms of resistance into vulnerabilities, representing a powerful approach for treating multidrug-resistant cancers [169]. Examples of cancer cell membrane-coated nanoparticles are presented in Table 7.

**Table 7:** Cancer cell membrane-coated nanoparticle strategies against multidrug resistance.^a^

Type of cancer	Nanoparticle used	1: Encapsulated drug, 2: Mechanism of action, 3: Experimental results, 4: Model, 5: MDR criterion, 6: Primary outcome measure	Ref.

gastric cancer	exosomes derived from sensitive cells (SGC-7901, MGC-803) loaded with miR-107	1: 5-fluorouracil (5-FU) + cisplatin (CDDP) 2: exosomes deliver miR-107 to resistant cells; miR-107 negatively regulates HMGA2; inhibition of HMGA2/mTOR/P-gp pathway → reversal of drug resistance 3: greater sensitivity of resistant SGC-7901/5-FU cells to 5-FU and CDDP; mechanism confirmed with exosome inhibitor (GW4869); luciferase assay validated miR-107/HMGA2 interaction 4: in vitro (SGC-7901/5-FU) 5: HMGA2/mTOR/P-gp pathway 6: 5-FU and CDDP sensitivity	[170]
lung cancer	cancer cell membrane-coated silica nanoparticles (CCM/SLI/miR-DOX) – A549/DOX-derived membrane on aminated silica core	1: doxorubicin (DOX) + miR-495 2: homotypic targeting via cancer cell membrane; miR-495 silences GCN5 (histone acetyltransferase regulating MDR1) → ↓ P-gp; sustained DOX release from silica core 3: 1.6-fold ↑ intracellular DOX; >50% ↓ P-gp vs controls; gradual volume decrease in 3D spheroids; near-total xenograft proliferation inhibition with negligible weight loss 4: in vitro + in vivo (A549/DOX xenograft) 5: GCN5 → ABCB1 transcription axis 6: P-gp expression; xenograft growth	[169]

^a^Abbreviations: 5-FU: 5-fluorouracil; CDDP: cisplatin; MSC-EVs: mesenchymal stem cell–derived extracellular vesicles; miR: microRNA; HMGA2: High Mobility Group AT-hook 2; TXNIP: thioredoxin-interacting protein; mTOR: mechanistic target of rapamycin; P-gp: P-glycoprotein (ABCB1); MDR: multidrug resistance; GC: gastric cancer.

**3.2.5 Comparative overview of nanocarriers for P-gp-mediated multidrug resistance.** To strengthen the comparative understanding of the nanocarrier systems previously discussed in this section and to provide clearer insight into their translational relevance in P-gp-mediated MDR, a structured comparison is presented in Table 8. This comparative synthesis highlights the distinct mechanisms of drug resistance modulation, key advantages, limitations, relative drug-loading capabilities, developmental status, and scalability potential of lipid-based, polymeric, and inorganic nanocarriers as reported in the literature covered in this review.

**Table 8:** Comparative overview of nanocarriers for P-gp-mediated multidrug resistance.^a^

Nanocarrier type: Lipid-based nanoparticles	

representative example(s) from the manuscript	functionalized liposomes (e.g., ApoA1-DOX liposomes targeting SR-B1); SLNs/NLCs for MDR drugs
mechanism for P-gp/MDR modulation	liposomes enable receptor-mediated endocytosis (e.g., SR-B1) increasing uptake; PEGylation prolongs circulation; SLNs/NLCs modulate release to reduce immediate efflux; lipid–polymer hybrids combine P-gp modulation (Pluronic/TPGS) with lipid biocompatibility
advantages	good biocompatibility; can load hydrophilic/hydrophobic drugs; extended circulation; established clinical familiarity for liposomes; NLCs mitigate crystallization and improve payload vs SLNs
limitations	potential premature leakage (liposomes) without proper design; stability/shelf-life depend on composition; SLNs limited for hydrophilic cargo; formulation tuning needed
drug-loading tendency (per manuscript discussion)	moderate to high for lipophilic drugs; NLCs > SLNs due to amorphous matrix; hybrids show high loading via dense lipid/polymer matrix
development stage reported	in vitro and in vivo MDR models (e.g., ApoA1-DOX liposomes achieved 79% tumor inhibition in MCF-7/ADR mice)
scalability notes	generally favorable: “low toxicity and ease of production” highlighted for LNPs; hybrids leverage scalable lipids + polymers

Nanocarrier type: Polymeric nanoparticles	

representative example(s) from the manuscript	stimuli-responsive micelles (TPGS-IDM; Pluronic hybrids); γ-PGA-*g*-PLGA nanocapsules (DOX + ICG); multistimuli polymers; DMA-NP cascade system
mechanism for P-gp/MDR modulation	micelles: trigger-controlled release (pH/redox/ROS), ATP depletion and direct P-gp inhibition (TPGS/Pluronic) → ↑intranuclear drug; nanocapsules: C-PEG disrupts lipid rafts & reduces P-gp; NIR photothermal burst ↑release; DMA-NPs: pH-charge reversal → size shrinkage → GSH-triggered release of non-P-gp substrate
advantages	high design flexibility; can co-deliver drugs + sensitizers/genes; precise TME-responsive release; strong preclinical efficacy (e.g., >80% tumor inhibition in MDR animals; 90% inhibition in A549/PTX with DMA-NP)
limitations	synthetic complexity; long-term stability/regulatory hurdles; dependence on heterogeneous TME triggers; external activation (e.g., NIR) in some designs
drug-loading tendency (per manuscript discussion)	often high for hydrophobic drugs (micellar cores); nanocapsules co-load drug + photothermal agent efficiently
development stage reported	robust in vitro/in vivo (MCF-7/ADR, A549/PTX); specific outcomes reported: e.g., 79% inhibition (lipid example) and 90% with DMA-NP in resistant lung cancer
scalability notes	promising but requires control of batch-to-batch and complex manufacturing, manuscript flags scalability as an ongoing challenge

Nanocarrier type: Inorganic nanoparticles	

representative example(s) from the manuscript	mesoporous silica nanoparticles (HPMSNs: DOX + siGCN5); gold nanoparticles (AuNPs)
mechanism for P-gp/MDR modulation	MSNs: high surface area/pore volume → high loading; dual pH/redox “gatekeeper” opens in endosomes; siGCN5 downregulates MDR1 → ↓ P-gp and ↑ DOX retention (77% tumor inhibition). AuNPs: size-engineered carriers; metallic nanoassemblies enable drug delivery + ROS/photothermal effects
advantages	very high loading (MSNs); precise surface functionalization; theragnostic potential for metals
limitations	potential bio persistence/toxicity concerns without proper design; careful size/ligand control needed for metals
drug-loading tendency (per manuscript discussion)	high for MSNs (large pore volume); tunable for AuNPs via surface ligands; both highlighted as efficient carriers in manuscript
development stage reported	in vitro/in vivo MDR models reported (e.g., HPMSNs: 77% inhibition, near-complete P-gp eradication in MCF-7/ADR xenografts)
scalability notes	feasible with established silica/metal chemistries, but translation requires addressing long-term safety and standardization

^a^Abbreviations: P-gp: P-glycoprotein; MDR: multidrug resistance; DOX: doxorubicin; ICG: indocyanine green; NIR: near-infrared; TPGS: ᴅ-α-tocopheryl polyethylene glycol succinate; ROS: reactive oxygen species; GSH: glutathione; C-PEG: cholesterol-polyethylene glycol; MSNs: mesoporous silica nanoparticles; HPMSNs: hollow periodic mesoporous silica nanoparticles; siRNA: small interfering RNA; EE: encapsulation efficiency; AUC: area under the concentration–time curve; NLCs: nanostructured lipid carriers; SLNs: solid lipid nanoparticles; LNPs: lipid nanoparticles; AuNPs: gold nanoparticles; TME: tumor microenvironment.

Overall, lipid-based nanocarriers are recognized for their biocompatibility and clinical familiarity, polymeric platforms offer versatile functionalization and controlled release under tumor-specific conditions, while inorganic systems provide high loading capacity and tunable physicochemical properties, though their long-term safety and regulatory acceptance remain under active investigation.

### 4 Challenges and future perspectives in overcoming ABC-mediated resistance

Despite the extensive library of nanocarriers designed to bypass or inhibit ABC transporters, the path from bench to bedside has proven far more difficult than early preclinical results suggested. Three bottlenecks, in particular, have repeatedly derailed otherwise promising strategies. The first is historical but instructive. The systemic toxicity of first-generation P-gp inhibitors (verapamil, cyclosporine A, and their contemporaries) was severe enough to render them clinically unusable, not because the science was wrong, but because the therapeutic window was simply too narrow to justify the risk to patients.

The second obstacle is more subtle and arguably more difficult to engineer around. Even when nanoparticles successfully enter the cell via endocytosis, bypassing membrane-bound P-gp entirely, the drug they carry does not remain safe once released. If that release occurs near the plasma membrane, the free drug finds itself in immediate proximity to the very efflux pumps it was meant to avoid. This phenomenon, sometimes referred to as the “release-efflux paradox”, represents one of the most underappreciated limitations in the field. The third challenge is perhaps the most humbling. Inhibiting P-gp alone is often not enough. Cancer cells respond by upregulating compensatory transporters, most notably BCRP and MRP1, essentially replacing one efflux mechanism with another and rendering single-target strategies insufficient.

Addressing these layered obstacles will require a genuine shift in design philosophy. Rather than continuing to optimize single-mechanism platforms, future nanotechnology must move toward multifunctional and stimuli-responsive systems capable of acting on several fronts simultaneously. The most viable paths forward include co-delivering genetic silencers such as siRNA alongside chemotherapeutics to suppress transporter expression at the source [34,39], incorporating materials like TPGS that actively deplete mitochondrial ATP and thereby starve the efflux machinery of the energy it needs to function, and developing morphology-transformable nanoparticles that physically restrict drug mobility within the cell, keeping the therapeutic payload where it needs to be long enough to do its job.

None of these approaches are simple, and no one will succeed in isolation. But taken together, they represent a more honest and sophisticated answer to a resistance mechanism that has, for decades, remained one step ahead.

## Conclusion

Nanotechnology has created new opportunities to address MDR in cancer, particularly through strategies aimed at modulating the function of P-glycoprotein. Diverse nanocarrier platforms, including lipid-based, polymeric, hybrid, and surface-functionalized systems, have demonstrated promising capacity to improve drug bioavailability, enhance tumor-selective delivery, and reduce transporter-mediated drug efflux.

This review highlights how advanced nanosystems can be rationally engineered to inhibit P-gp activity, bypass membrane efflux through endocytosis or tumor-responsive release, and suppress transporter expression through gene-silencing approaches. Collectively, these strategies have shown encouraging preclinical outcomes, including enhanced intracellular drug accumulation, restored chemosensitivity, and reduced systemic toxicity in resistant tumor models.

Despite these advances, clinical translation remains challenging. Key limitations include nanoparticle instability during storage or circulation, biological variability among patients and tumor types, manufacturing scalability, regulatory complexity, and the lack of standardized efficacy metrics across studies. Addressing these barriers will require interdisciplinary collaboration, robust translational design, and carefully structured clinical evaluation pathways.

In addition, future platforms should consider that repeated administration of nanocarriers may itself trigger adaptive biological responses. Phenomena such as accelerated blood clearance (ABC), increased opsonization, complement activation, and enhanced macrophage uptake have been reported in some long-circulating systems, particularly PEGylated formulations. These responses may reduce therapeutic performance over time and represent an emerging challenge related to carrier clearance rather than classical drug efflux. Therefore, immunogenicity, stealth durability, and long-term biodistribution should be considered critical design parameters in next-generation MDR nanomedicine.

Among currently explored approaches, polymeric micelles and ligand-functionalized liposomes appear especially promising because of their manufacturability, favorable safety profiles, and capacity for co-delivery of chemotherapeutic agents with P-gp modulators or nucleic acid payloads. However, no single platform has yet emerged as universally superior.

Overall, nanocarrier-based modulation of P-glycoprotein represents a promising translational strategy. Its ultimate clinical impact will depend on whether sophisticated preclinical systems can be converted into reproducible, scalable, and patient-benefiting therapies in real oncological settings.

## Expert Commentary: Nanotechnology as a Paradigm Shift in Oncology

The evolving field of nanomedicine presents several promising avenues to enhance the efficacy and clinical applicability of nanoparticle-based strategies targeting MDR.

### Integration of immunotherapy and modulation of the tumor microenvironment

The integration of nanoparticle-mediated drug delivery with immunotherapeutic agents or modulators of the tumor microenvironment may enhance therapeutic efficacy through synergistic effects. Nanoparticles may be engineered to co-deliver immune checkpoint inhibitors with chemotherapeutics while concurrently downregulating P-gp expression.

#### Artificial intelligence and computational modeling

AI-based tools facilitate the efficient design of nanocarriers by forecasting optimal physicochemical properties, biodistribution profiles, and therapeutic windows. Models utilizing machine learning, when applied to pharmacokinetic and toxicity datasets, have the potential to minimize trial-and-error in the optimization of nanoparticles.

#### Personalized nanomedicine

Future strategies may concentrate on customizing nanoparticle formulations to align with the specific genetic and phenotypic characteristics of tumors. This involves the utilization of patient-derived organoids or tumor-on-chip systems to assess and confirm nanoparticle efficacy ex vivo prior to clinical application.

#### Biomimetic and cell-membrane coated systems

Biomimetic nanoparticles, including those coated with cancer cell membranes, red blood cells, or exosomes, demonstrate potential for immune evasion and targeted delivery. Their capacity to replicate natural cellular interactions may enhance tumor accumulation and minimize off-target effects.

#### Clinical trial design and regulatory harmonization

It is essential to develop rigorous clinical trials that incorporate clearly defined endpoints for multidrug resistance reversal and drug accumulation. Regulatory frameworks must evolve to address the complexities of multifunctional nanomedicines, ensuring safety and promoting innovation.

#### Long-term toxicity and biodegradation investigations

Thorough examinations of long-term behavior, biodegradation, and removal of nanoparticles are crucial for ensuring safety in chronic or repeated dosing situations. Future investigations should examine the clearance pathways of nanoparticles and the potential for accumulation in non-target tissues. Implementing these guidelines will enhance the design and functionality of nanoparticle systems, advancing the field toward clinically viable solutions for addressing multidrug resistance in cancer therapy. Nanocarrier-mediated reversal of MDR offers a promising opportunity to synergize with immune checkpoint blockade. By enhancing intracellular drug accumulation and promoting immunogenic cell death, P-gp-inhibitory nanosystems increase the release of tumor-associated antigens and facilitate T-cell activation. Furthermore, cancer cell membrane-coated nanoparticles can act as autologous vaccines that simultaneously resensitize tumors to chemotherapy and potentiate PD-1/PD-L1 blockade responses. This dual strategy represents a relevant frontier in immuno-nanomedicine, integrating MDR reversal with sustained antitumor immunity.

Although some of the in vitro results are quite promising, numerous translational hurdles remain, including biological, manufacturing, and regulatory challenges. The most common biological problems are the high intratumoral heterogeneity in P-gp expression, which leads to uneven drug distribution, resulting in incomplete reversal of multidrug resistance and tumor recurrence [113]. Furthermore, multifunctional nanoparticles can trigger immune responses, leading to their elimination by the system and reducing their bioavailability. Additionally, nanoparticles can accumulate in non-target organs, causing systemic toxicity [171]. However, new ideas are being proposed to overcome these challenges, such as designing nanocarriers with characteristics more sensitive to the microenvironment, not only in terms of pH but also through enzyme activation [154]. Furthermore, surface modification with zwitterionic polymers is being explored to evade immunological detection, as well as the prediction of pharmacokinetic profiles using artificial intelligence for site-specific targeting [172].

Another challenge lies in the scalability of laboratory production. Synthesis is not easily scaled up due to inconsistencies in the size and distribution of nanocarriers in large batches. Furthermore, multifunctional designs increase complexity and costs, limiting the feasibility of large-scale preclinical validation [173]. Therefore, options such as the adoption of robotic platforms to enable automated production and modular design approaches are being studied to avoid high costs and improve scalability.

Finally, regulatory hurdles delay the translation of research. Agencies such as the FDA and EMA lack specific regulations for nanotechnology, making it difficult to classify hybrid nanocarriers as drugs or devices [174]. Furthermore, the complex assessment of the long-term toxicity of these nanocarriers requires extensive preclinical data, which frequently reveals unexpected risks, leading to the discontinuation of studies [140]. That is why the FDA is currently conducting a Nanotechnology Regulatory Science Program, in addition to promoting phased testing and the use of alternative models [175].

The reproducibility and efficacy of P-gp-targeted nanocarriers largely depend on their physicochemical characteristics and manufacturing consistency. Critical quality parameters such as particle size, polydispersity index (PDI), zeta potential, encapsulation efficiency, drug release profile, and colloidal stability determine both pharmacokinetic behavior and therapeutic outcome. A low PDI (<0.2) reflects a homogeneous nanoparticle population and predictable biodistribution, whereas adequate zeta potential values (±20–30 mV) prevent aggregation and enhance circulatory stability. Likewise, maintaining a narrow size distribution (100–200 nm) favors tumor accumulation through the enhanced permeability and retention effect. Controlled and sustained drug release supports prolonged retention in circulation and consistent intracellular delivery, correlating directly with improved efflux inhibition and drug sensitivity restoration described throughout this review.

Furthermore, reproducibility across experimental batches remains a central requirement for clinical translation. The integration of standardized characterization protocols, including physicochemical evaluation, release kinetics, and long-term stability, ensures comparability among different nanoplatforms and validates their performance in preclinical assays. These aspects also complement the discussion of translational challenges by linking formulation parameters to biological reliability. The inclusion of these quality metrics strengthens reproducibility, safety, and overall therapeutic efficiency of P-gp-modulating nanomedicines.

## Data Availability

Data sharing is not applicable as no new data was generated or analyzed in this study.
